# The Impact of Oxidative Stress in Human Pathology: Focus on Gastrointestinal Disorders

**DOI:** 10.3390/antiox10020201

**Published:** 2021-01-30

**Authors:** Rosa Vona, Lucia Pallotta, Martina Cappelletti, Carola Severi, Paola Matarrese

**Affiliations:** 1 Center for Gender-Specific Medicine, Istituto Superiore di Sanità, Viale Regina Elena 299, 00161 Rome, Italy; paola.matarrese@iss.it; 2 Department of Translational and Precision Medicine, Sapienza University of Rome, Viale del Policlinico, 155, 00161 Rome, Italy; lucia.pallotta@uniroma1.it (L.P.); cappelletti.1942536@studenti.uniroma1.it (M.C.); carola.severi@uniroma1.it (C.S.)

**Keywords:** oxidative stress, gastrointestinal diseases, gastrointestinal muscle inflammation, antioxidants

## Abstract

Accumulating evidence shows that oxidative stress plays an essential role in the pathogenesis and progression of many diseases. The imbalance between the production of reactive oxygen species (ROS) and the antioxidant systems has been extensively studied in pulmonary, neurodegenerative cardiovascular disorders; however, its contribution is still debated in gastrointestinal disorders. Evidence suggests that oxidative stress affects gastrointestinal motility in obesity, and post-infectious disorders by favoring the smooth muscle phenotypic switch toward a synthetic phenotype. The aim of this review is to gain insight into the role played by oxidative stress in gastrointestinal pathologies (GIT), and the involvement of ROS in the signaling underlying the muscular alterations of the gastrointestinal tract (GIT). In addition, potential therapeutic strategies based on the use of antioxidants for the treatment of inflammatory gastrointestinal diseases are reviewed and discussed. Although substantial progress has been made in identifying new techniques capable of assessing the presence of oxidative stress in humans, the biochemical-molecular mechanisms underlying GIT mucosal disorders are not yet well defined. Therefore, further studies are needed to clarify the mechanisms through which oxidative stress-related signaling can contribute to the alteration of the GIT mucosa in order to devise effective preventive and curative therapeutic strategies

## 1. Introduction

Oxidative stress in living organisms results from the imbalance between the production of reactive oxygen species (ROS) and the ability to neutralize them. The disparity between excessive reactive molecules and weak endogenous defense leads to damage to cell structures and molecules such as lipids, proteins, and DNA, ultimately contributing to the pathogenesis of a wide range of diseases. ROS, when available in appropriate low amounts, act as signal transduction molecules driving cell activities and also provide cell protection [1]. On the other hand, if generated in excess, as in inflammation, ROS can trigger the production of additional highly reactive species [2]. Crucially is the oxidative modification of key enzymes or regulatory sites, whose redox modification triggers cell signaling alteration and programmed cell death. Oxidative stress and inflammation are closely linked. Oxidative stress can cause inflammation and this, in turn, induces oxidative stress generating a vicious circle [3,4] that results in cell damage, which promotes a pro-inflammatory environment [5].

**Figure 1:**
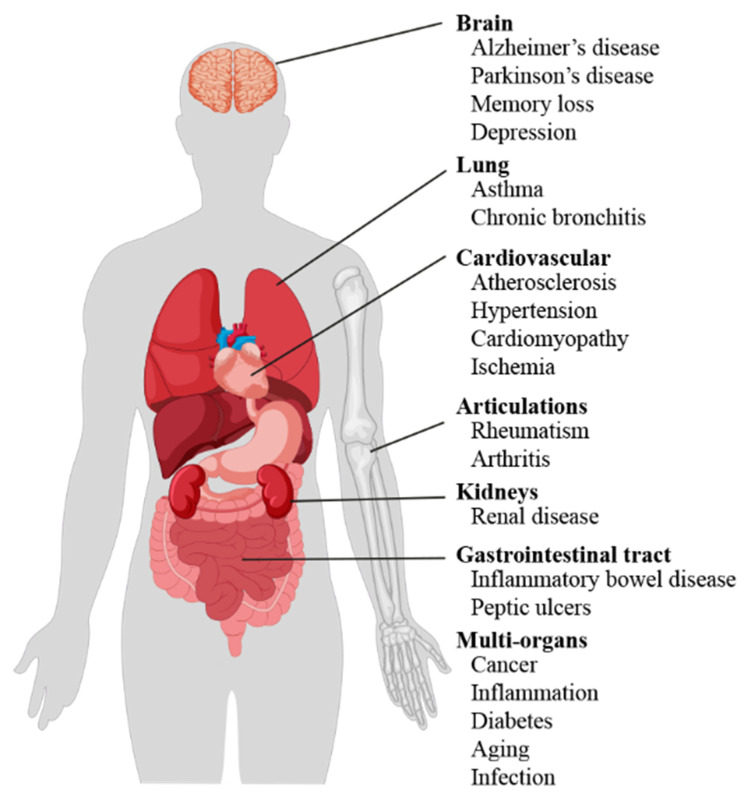
Scheme of oxidative stress-induced diseases in humans.

Literature data confirm the key role of oxidative stress in etiology of numerous and different diseases (Figure 1), including metabolic syndrome [6], atherosclerosis [7], cardiovascular disease [8,9], cancer [10,11], neurodegenerative disorders [12,13] diabetes [14], infertility [15], renal diseases [16], and gastrointestinal and hepatic diseases [17].

Being involved in the absorption of nutrients and in the immune response, the gastrointestinal tract (GIT) plays a key role also in the production of ROS. Several evidences highlight how the pathogenesis of various GIT diseases, including colorectal and gastric cancers [18,19,20], inflammatory bowel disease (IBD) [21,22], and peptic ulcers [23], is due, at least in part, to oxidative stress.

The GIT tissue is structured into four layers: the mucosa (epithelium, lamina propria, and muscular mucosae), the submucosa, the muscularis propria (inner circular muscle layer, intermuscular space, and outer longitudinal muscle layer), and the serosa.

The intestinal epithelia are exposed continuously to a wide variety of potentially harmful substances and act as a selective barrier between the tissues and luminal environment of the GIT. There are several stressors, which induce the generation of free radicals and result in oxidative stress and GIT inflammatory responses involving the epithelium and immune/inflammatory cells [24]. Although there is enough information on the role played by oxidative stress in the damage of intestinal mucosa, little is known about the involvement of the surrounding muscle layers.

Knowledge of the biochemical mechanisms underlying the alterations induced by oxidative stress at the GIT level, as well as of the physiological responses of the different GIT layers to such stress, is mandatory to better understand either pathogenesis of GIT diseases or to develop new and more effective therapeutic strategies.

This review summarizes the current understanding of the role of oxidative stress in GIT pathophysiology, also discussing the specific molecular mechanisms involved, focusing particular attention on the implication of the muscular layers of the GIT.

## 2. Oxidative Stress

Oxidative stress occurs when, in tissues and organs, the formation of highly reactive molecules e.g., ROS, reactive nitrogen species (RNS), and reactive sulfur species (RSS), overcome the endogenous antioxidant defense system capacities, leading to cellular damage and dysfunctions that result in a wide range of diseases. The reactive species are constantly generated within cells at low concentrations as a result of normal metabolic processes. They can also results from the exposure to external factors like radiation (X-rays and UV), ozone, air pollutants, cigarette smoke, bacteria, viruses, drugs, etc. [25], or as the outcome of an acute or chronic cellular stress. The reactive species can be free radicals and non-radical oxidants. The free radicals are unstable because of unpaired electrons presence in their outer electron orbit. Since free radicals are highly unstable and reactive, tend to neutralize themselves by reacting with other molecules causing their oxidation [26]. Therefore, by reacting with important biological molecules, including DNA, lipids and proteins, they can cause damage on various levels [27]. Proteins, being among the main components of the cells, represent major targets for free radicals [28]. Free radicals can induce some protein modifications, i.e., unfolding or alteration of protein structure, most of which, fortunately, are essentially harmless events [29]. While the reversible oxidative changes are involved in the regulation of protein activity, irreversible protein changes can lead to their inactivation with consequent lasting harmful cellular effects [29].

**Figure 2:**
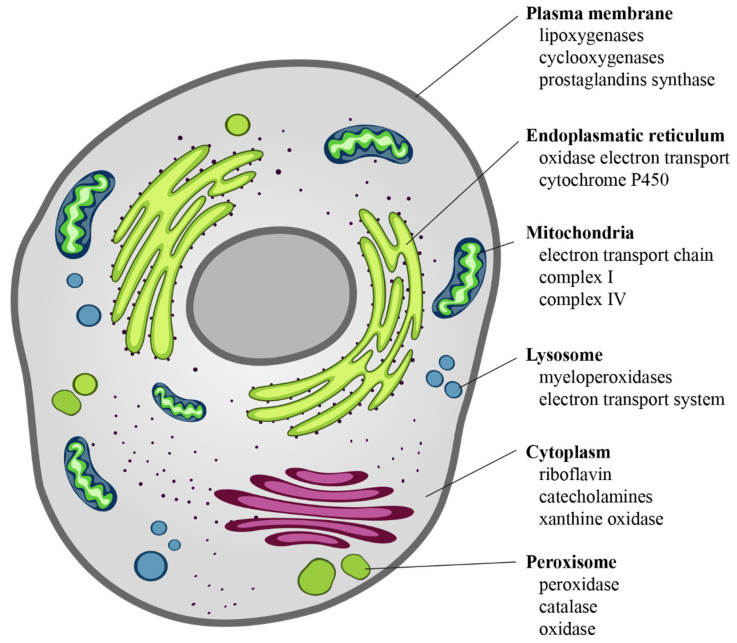
Cellular sources of ROS. ROS are the “by-products” of electron transfer reactions. The major source of ROS is the mitochondrial electron transport chain, followed by the NADPH oxidases present on either side of the plasma membrane. In the smooth endoplasmic reticulum, we find cytochrome P-450 and b5 families, which are responsible for a series of reactions to detoxify fat-soluble drugs and harmful metabolites. Peroxisomes, through their oxidases, are a significant source of total cellular H_2_O_2_ production. Moreover, they are responsible for dismutation of H_2_O_2_ to H_2_O and O_2_, and of fatty acids β-oxidation. Other enzymes, present free in the cytoplasm, such as xanthine oxidase, aldehyde oxidase, flavoprotein dehydrogenase, and tryptophan dioxygenase can produce ROS during catalytic cycling.

The intracellular sources of chemical reactive species are mainly mitochondria, endoplasmic reticulum, lysosomes, peroxisomes, cytosol, and plasma membrane [30] (Figure 2). ROS derive from the chemical reduction of molecular oxygen and, among the main ones, we find: the free radicals, such as superoxide anion radical (O_2_^•^^−^), hydroxyl radical (^•^OH), as well as non-radical oxidant, such as hydrogen peroxide (H_2_O_2_) and hypochlorous acid (HClO) [31]. Among the RNS, the major players are peroxynitrite radical (ONOO^−^), ozone, and nitric oxide (^•^NO) [32]. The new identified RSS include thiol radical (RS), and RSS both formed by the reaction between ROS and thiols. Similarly, RSS include radical species, such as (RSR^•^), glutathionyl radical (GSSG^•^), and non-radicals ones, such as reactive sulfane species (RSR), reactive sulfur substances (SO_2_, SO_3_), etc. [33,34]. In particular, RSS are able to trigger both oxidation and reduction reactions with particular tropism for sulfur-containing molecules, such as peptides and proteins [33,34].

The most important sites of ROS production are the enzymes of the mitochondrial electron transport respiratory chain. Other enzymes catalyze chemical reactions contributing to the ROS formation, among them the homologs of nicotinamide adenine dinucleotide phosphate (NADPH) oxidase, phospholipase A2 (PLA2), uncoupled nitric oxide synthase (NOS) as well as cyclooxygenases (COX), xanthine oxidase (XO), lipoxygenases (LOXs), glucose oxidase, and myeloperoxidase (MPO) [24,35,36]. NADPH oxidase, an enzyme present in the plasma membrane, was initially discovered in the phagosomes of macrophages, neutrophils, and monocytes. There are six homologs of NADPH oxidase, NOX1, NOX3-5, and dual oxidase (DUOX) 1 and 2, with several intracellular localizations [37]. Literature data showed that NOX1 and DUOX2 have significant roles in *Helicobacter pylori*-induced gastric inflammation, inflammatory bowel disease (IBD), and tumors [24,38]. XO is present in the cytoplasm and also on the outer surface of the plasma membrane; it is mainly expressed in the liver and small intestinal mucosa within the gastrointestinal tract [39]. LOXs are non-heme iron enzymes that can generate ROS catalyzing oxidation of arachidonic acid (AA). MPO is a heme-enzyme localized in lysosomes of neutrophils, macrophages, and monocytes. Several data demonstrate that MPO activity is increased in inflamed mucosa in ulcerative colitis and also in *H. pylori*-infected subjects [24], playing a role in the development of *H. pylori*-induced atrophic gastritis. The chronic oxidative stress related to ulcerative colitis and *H. pylori*- infection could also lead to cancer, often associated with these diseases [40,41]. NOS is a heme-containing monooxygenase that generates NO. There are three different isoforms of NOS: neuronal NOS (nNOS), endothelial NOS (eNOS), and endotoxin or cytotoxin-inducible NOS (iNOS) [42]. In GIT, NOS expression and activity are very important because the generation of NO maintains normal functions of mucosa and plays a cytoprotective role. Indeed, NO regulates blood flow, epithelial secretion, and barrier function of gastric mucosal [43] and represents one of the main enteric neurotransmitters mediating GI muscle relaxation [43,44]. However, NO can also have deleterious effects, and iNOS expression was found increased in chronic ulcerative colitis and peptic ulcer patients [24]. COX enzyme releases AA from the membrane phospholipids and catalyzes AA conversion to prostanoids. COX has two isoforms, COX-1 and COX-2, both of which are expressed in normal human gastric mucosa. COX-1 is constitutively expressed, while COX-2 is induced by inflammation and tumorigenesis [45]. COX-2 has also been reported to have cytoprotective functions in human colon and gastric cancer cells where it was induced during high osmotic stress [46]. Therefore, reactive oxygen species, including oxygen free radicals, are generated by the activity of several types of oxidases. Initially, O_2_ is reduced by the addition of electrons, thereby producing O_2_^•^^−^ that can react with other endogenous molecules to generate secondary oxidizing molecules, such as ONOO^-^. Thereafter, the reduction of O_2_^•^^−^ leads to the by-product H_2_O_2_ that is characterized by a long life span and relative stability. The latter is enzymatically converted into water and O_2_, or possibly into different metabolites, thus extinguishing the radical cascade [6].

Both O_2_^•^^−^ and H_2_O_2_ are also important signaling molecules, particularly in vascular smooth muscle cells where they can trigger specific biochemical pathways that regulate the defense mechanisms following exposure to oxidative stress. At the center of these pathways are for example mitogen-activated protein kinases, and tyrosine kinases, and transcription factors [47]. Particularly transcription factors, such as activator protein-1 (AP-1), NF-κB, and/or NF-E2-related factor (NRF2) have been reported to also participate in redox-modulated cell signaling [48,49].

## 3. Antioxidants

If the body’s antioxidant defense system fails to neutralize the excess free radicals, the imbalance between oxidants and the defense system can lead to pathological conditions, including cancer [10,11], cardiovascular disease [7,8], neurodegenerative disorders [12,13], atherosclerosis [7], and others. Halliwell and Gutteridge defined antioxidants as “*any substance that delays, prevents or removes oxidative damage to a target molecule*” [50,51,52]. All living organisms are endowed with endogenous antioxidant defenses capable of contrasting and removing reactive chemical species. However, these defenses are insufficient to totally remove reactive species and completely prevent oxidative damage to cells, tissues, and organs [4]. The endogenous antioxidants can act at various levels: blocking the formation of radicals, neutralizing them by oxidizing themselves, or delaying the oxidation reactions of other molecules. Moreover, some antioxidants, acting as metal chelators, transform metal pro-oxidants into more stable chemical forms.

**Figure 3:**
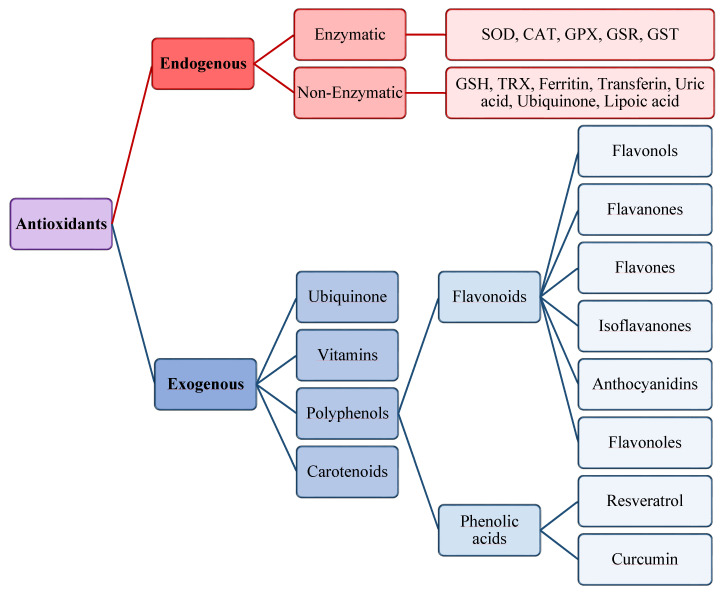
Scheme of endogenous and exogenous antioxidants. SOD, Superoxide dismutase; CAT, Catalase; GPX, Glutathione peroxidase; GSR, Glutathione reductase; GST, Glutathione transferase.

The antioxidants were be classified by Gutteridge and Halliwell into three categories: primary, secondary, and tertiary antioxidants, on the bases of their mechanism of action [51]. Primary antioxidants inhibit oxidant formation; secondary antioxidants function as scavengers of ROS, and tertiary antioxidants repair the oxidized molecules. Currently, antioxidants are substantially classified as enzymatic or non-enzymatic (Figure 3).

### 3.1. Enzymatic Antioxidants

Among the enzymatic antioxidants that contribute to the defense against the reactive species, we find catalase (CAT), superoxide dismutase (SOD), glutathione peroxidase (GPX), and glutathione reductase (GSR). Enzymatic antioxidants have both primary and secondary defense functions and represent an endogenous antioxidant system. Glutathione peroxidase, SOD, and catalase are the primary defense that prevents the formation or neutralize reactive species [53]. In particular, SOD and catalase provide major antioxidant defenses against ROS.

SOD catalyzes the dismutation of O_2_^−^ into O_2_ and H_2_O_2_. In humans beings are present three isoforms of SOD [54]: cytosolic copper and zinc-containing enzyme (Cu-Zn-SOD), present in the mitochondrial inter-membranous space; manganese-requiring mitochondrial enzyme (Mn-SOD), present in the mitochondrial matrix; and extracellular Cu-Zn containing SOD (EC-SOD) [55]. H_2_O_2_ not scavenged by GPX located at the level of the mitochondrial matrix crosses the mitochondrial membrane towards the cytosol, where it can be scavenged either by cytosolic Cu-Zn-SOD or CAT [56]. Increased levels of all three SOD isoforms are present in intestinal tissues from IBD patients, particularly in the epithelium [57], and in patients with ulcer healing [58]. Increased expression of Mn-SOD is associated with colorectal cancer, and it was also found increased in normal mucosa of gastric adenocarcinoma as well as in squamous cell oesophageal carcinoma tissues [59]. Moreover, a gastrointestinal mucosal injury could be prevented by the presence of SOD [60].

CAT, present mainly in peroxisomes, dismutates H_2_O_2_ to H_2_O and O_2_ [61]. In humans, CAT has been found virtually in all organs although it is produced largely in liver, kidney, and erythrocytes. Lower catalase activity was observed in colorectal cancer [62], gastric adenocarcinoma, in *H. pylori*-infected stomach [62], and in Crohn’s disease [63].

GPX converts glutathione (GSH) into its oxidized form (GSSG), reduces H_2_O_2_ to H_2_O, and lipid hydroperoxides (ROOH) to the corresponding stable alcohols. The GPX reaction is paired to glutathione reductase (GSR), which maintains reduced glutathione (GSH) levels. GSR, GPX, and glutathione S-transferases (GST), form the glutathione system that in the GIT mucosa acts as an antioxidative barrier. This enzyme, generating GSH, is important for the protection of cell membranes, red blood cells, and hemoglobin to oxidative stress [64]. GPX is found in the mitochondria, cytoplasm, and extracellular space [65], and protects cells from the harmful consequences of peroxide decomposition. In humans, there are eight isotypes of GPX. While GPX1 is ubiquitous, GPX2 is specific for the gastrointestinal tract and protects the gut against the absorption of dietary hydroperoxides [66]. Moreover, GPX2 defends the gastrointestinal tract against ROS derived from gut inflammation associated with commensal bacteria [67]. Importantly, glucose-6-phosphate dehydrogenase, while not directly neutralizing the radicals, can be considered an antioxidant enzyme. This oxidoreductase maintains the level of NADPH, thus helping to keep glutathione in its reduced state (GSH) [68] and creating a reducing environment [53].

Thioredoxin reductase (TrxR) together with thioredoxin (Trx) forms the thioredoxin system. There are three TrxR isoforms: TrxR1 found in the cytoplasm, TrxR2 in mitochondria, and TrxR3 present only in specialized tissues. TrxR, by transferring reducing equivalents from NADPH to thioredoxin, keeps it in its reduced form [69]. It has been shown that a compensatory upregulation of TrxR mRNA in gastrointestinal cancer was induced by oxidative stress provoked by bile acids [70].

### 3.2. Non-Enzymatic Antioxidants

Among the endogenous non-enzymatic antioxidants, there are glutathione and Trx. Glutathione is ubiquitously expressed mostly in its reduced form, GSH. Glutathione is a strong antioxidant, certainly one of the most important among those that the body can produce. Relevant is its action against both free radicals and molecules such as hydrogen peroxide, nitrites, nitrates, benzoates, and others. An important element for its functioning is NADPH. In fact, this molecule, a derivative of vitamin PP (nicotinic acid), functions as a redox cofactor of the enzyme GSR, which reduced glutathione (GSH) from oxidized glutathione (or GSSG) through electrons transferred from NADPH to GSSG [53].

Trx contains two free sulfhydryl groups of two cysteine residues. It is involved in the biosynthesis of deoxynucleotides, since it reduces the oxidized ribonucleotide reductase by yielding their hydrogens to the two oxidized sulfhydryl groups of the ribonucleotide reductase. Trx is present in the cytoplasm, membranes, and mitochondria but also in the extracellular space [71]. It showed a cytoprotective action in various inflammatory conditions, and was found to regulate the activity of redox-sensitive transcription factors, which are part of the antioxidant defence system.

Ubiquinone, also known as CoQ, is a lipophilic molecule existing in three different redox states: fully oxidized, partially reduced (ubisemiquinone), and fully reduced (ubiquinol) [72]. It is found in the plasma membrane and in several intracellular membrane including mitochondrial ones where it plays a key role in energy production and ROS generation. In its fully reduced form, CoQ is a potential antioxidant. Experimental studies have shown a protective role of ubiquinone against protein carbonylation and oxidative damage to DNA [73,74]. Furthermore, it is also been shown that ubiquinone can prevent peroxidative damage to membrane phospholipids [75] and regenerate other powerful antioxidants, such as α-tocopherol and ascorbate, by recycling them back to their reduced active forms, thus increasing resources cellular antioxidants [76]. These properties make ubiquinone suitable as a food supplement to improve cellular bioenergetics and to counteract some age-related diseases [76].

Activator protein-1 (AP-1), nuclear factor kappa B (NF-κB), and nuclear factor (erythroid-derived 2)-like 2 related factor (NRF2) are three transcription factors that have been reported to be involved in redox-modulated signaling pathways. Indeed, oxidative stress up-regulates NF-κB activity, and AP-1 and NRF2 activation depends on the environmental and/or intracellular redox state. Under normal conditions, NRF2 is found blocked in the cytosol by its inhibitor, KEAP1. Oxidative modification of KEAP1 and NRF2 phosphorylation result in the release of NRF2 from KEAP1 [77] and its translocation into the nucleus, where it binds with antioxidant response elements involved in activation of gene expression, thereby protecting cells from free radical damage. Therefore, NRF2, through its interaction with antioxidant response element (ARE), is able to modulate the expression of defensive genes coding detoxifying enzymes and antioxidant proteins [78].

### 3.3. Exogenous Antioxidants

Besides the endogenous enzymatic and non-enzymatic antioxidant defenses, other antioxidants are also utilized by the body, which must necessarily be introduced through diet and for this reason are defined exogenous. In addition to endogenous antioxidants, exogenous ones act through different mechanisms and in different cellular compartments. They are mainly free radical scavengers: they neutralize free radicals, repair oxidized membranes, and decrease reactive oxygen species production [79]. Among the exogenous antioxidants, we find: vitamins (A, C, E, and K), enzyme cofactors (Q10), nitrogen compounds (uric acid), minerals (zinc, Zn and selenium, Se), and polyphenols (flavonoids and phenolic acid) [80]. Metals such as manganese, zinc, copper, iron, and selenium up-regulate the catalytic activity of antioxidant enzymes [81]. It has been indicated that an inadequate dietary intake of these trace minerals may compromise the effectiveness of antioxidant defense mechanisms [82].

Exogenous antioxidants have generated a growing interest in preventing or reducing oxidative stress. In fact, many epidemiological researches have highlighted how the use of foods containing antioxidants and scavengers has a potential protective effect against the disorders caused by oxidative stress [83,84,85,86,87]. By increasing the body’s natural antioxidant defenses, or by supplementing with dietary antioxidants, various chronic diseases can be prevented or their progression can be slowed down. Natural antioxidants such as flavonoids, tannins, and polyphenols act by donating electrons to intermediate radicals and play a role in the inhibition of lipid peroxidation. For example, vitamin E, particularly its active form α-tocopherol, protects cells from lipid peroxidation and helps in the prevention of chronic diseases associated with oxidative stress [88,89].

The antioxidant phytochemicals contained in vegetables and fruits are considered a benefits to the health. Indeed, several studies demonstrated that they have antioxidant abilities both in vitro and in vivo [90,91]. Moreover, literature data highlighted that antioxidant phytochemicals can also have anti-inflammatory action [92]. In fact, natural compounds such as curcumin, resveratrol, and anthocyanins could reduce inflammation via inhibition of prostaglandin production, NF-κB activity, and specific oxidative enzymes, as well as by increasing anti-inflammatory cytokine (e.g., IL-10) or decreasing pro-inflammatory cytokine (i.e., IL-1β) production [93,94].

## 4. Available Methods to Assess Oxidative Stress in Clinical and Research Approaches

Currently, one of the most interesting challenges in studying oxidative stress is identifying biomarkers that can be used in clinical diagnostics. According to the World Health Organization, a biomarker is “any substance, structure or process that can influence or predict the incidence of outcomes or diseases and be measured in the body or its products” [95].

Although oxidative stress markers can often be measured easily and scientific evidence suggests that oxidative stress can influence the onset and evolution of numerous diseases, they are not always considered clinically relevant biomarkers. In fact, a biomarker is clinically useful when it is specific for certain pathology (diagnostic marker), or has value in predicting the evolution of the disease or is related to the degree of disease (prognostic marker). Furthermore, to be clinically useful, a biomarker must also be reasonably stable, present in easily accessible tissue and cost-effective to evaluate. Venous blood and urine are commonly used in clinical practice to detect oxidative stress, but in particular pathological conditions measurements of oxidative stress can also be conducted in cerebrospinal fluid [96], and other tissues [97,98].

In 2015, Frijhoff and colleagues examined the biomarkers used to assess oxidative stress, focusing on those most suitable for clinical and diagnostic use [99]. Possible markers of oxidative stress include the amount production of ROS produced, some downstream effects induced by ROS, and antioxidant defenses. Direct quantification of ROS, due to their short half-life, is quite a daunting task. Possible methods of measurement in biological systems include electron spin resonance, fluorescence magnetic resonance, and mass spectrometry techniques [100,101], but their use was limited to cell cultures and other in vitro applications. Flow cytometry is the most widely used method in clinical practice and research. In recent years, many fluorescent probes have been developed for the detection of reactive species, with a different degree of specificity and sensitivity [102]. The most commonly used fluorescent probes in clinical diagnostics for the detection of reactive species in cells by flow cytometry are shown in Table 1.

In addition to direct measurement of free radicals, a different approach is to measure reaction products of biological molecules with oxidizing species that may indicate systemic or tissue-specific oxidative stress. Indeed, as previously mentioned, molecules such as lipids, DNA, and proteins can be modified by the interaction with ROS, producing stable products that can be easily quantified [27,28]. Table 2 summarizes the main oxidation products of biological molecules used as markers of oxidative stress. Among the lipid oxidation products, useful as markers of oxidative stress, and involved in a variety of chronic diseases, trans-4-hydroxy-2-nonenal (4-HNE) and malondialdehyde (MDA) are the most studied. Several methods are available for the detection of both MDA and 4-HNE but the most reliable are the immunohistochemical and ELISA methods, in particular for HNE [103]. F2-isoprostanes (F2-IsoPs) are other markers used for the evaluation of oxidative stress in vivo. F2-IsoPs are formed in lipid membranes as a reaction between polyunsaturated fatty acids and ROS, and are therefore released in free form by the action of phospholipases. The measurement of F2-IsoP in biological fluids, as well as in the condensation of the breath, can provide an estimate of the systemic oxidative stress, while the measurement of esterified F2-IsoP in specific tissues can quantify a circumscribed oxidative stress. The most reliable methods for their quantification, the gas/liquid chromatography coupled with the mass spectroscopy techniques (HPLC/GC-MS), are laborious and require specialized and expensive instrumentation [104], while commercial immunoassays are often less reliable [105].

The nucleic acids DNA and RNA also represent a target of oxidative stress, particularly in their guanine bases. Oxidized nucleosides are excreted in the urine and their quantification can be interpreted as the cumulative total body oxidative stress. They are therefore able to provide information on systemic oxidative stress. Several commercial ELISAs are available to measure DNA damage with 7,8-dihydro-8-bone-2′-deoxyguanosine (8oxodG) and RNA damage with 7,8-dihydro-8-bone-2′-guanosine (8oxoGuo). The clinical use of chromatography coupled with mass spectrometry to detect oxidized nucleosides can be excessively expensive. Nucleic acid oxidation products have also been shown to predict the development of certain diseases [106].

Protein carbonyl groups originate from the oxidative cleavage of proteins by various mechanisms. They are usually detected after derivatization with 2,4-dinitrophenylhydrazine (DNP). The resulting carbonyl-2,4-dinitrophenylhydrazine adduct [107] can be detected spectrophotometrically, by ELISA, or by immunohistochemical, cytochemical, and western blot techniques using specific anti-DNP antibodies [108,109]. For clinical use, ELISA (commercially available) and HPLC tests are the only applicable methods. The functional groups of proteins can react with different molecules oxidized by ROS, such as polyunsaturated fatty acids and carbohydrates, generating respectively advanced peroxidation (ALE) and advanced glycation (AGE) end products [110,111]. This physiological process is particularly accentuated in conditions of hyperglycemia, hyperlipidemia, and oxidative stress. Protein adducts can be identified by mass spectrometry-based techniques, but their use is still limited in routine clinical analysis [112]. Furthermore, specific antibodies or spectrofluorimetric measurements based on the fluorescent properties of adducts are available [113,114]. Low-density oxidized lipoproteins (oxLDL), present in peripheral blood, are biomarkers of oxidative stress in cardiovascular disease, atherosclerosis, diabetes and obesity [115,116]. OxLDLs are measured in plasma or isolated LDLs by immunological methods using specific antibodies.

The redox state of cells, tissues or the whole organism can also be assessed by measuring the change in antioxidant defense systems in response to increased oxidative stress. The main players in this context include cysteine protein residues, the pool of antioxidants, ROS-generating enzymes, and transcription factors involved in their regulation (Table 3) [117].

The cysteine residues exposed on the cell surface are particularly sensitive to oxidation by ROS. Once oxidized, they can be reduced again by the reaction with GSH and/or by specific enzymatic activities (e.g., by thioredoxins, glutaredoxins and isomerase of the disulfide protein) [118,119]. The measurement of GSH, GSSG and their ratio in the blood was considered an index of the systemic redox status [120]. Different methods have been used to determine GSH in biological samples (spectrophotometry, HPLC, capillary electrophoresis, nuclear magnetic resonance and mass spectrometry) [120]. As mentioned above, Nrf-2 regulates the cellular response to oxidative stress by promoting the transcriptional activation of genes containing antioxidant response elements (ARE) in their promoter regions. Among these, the genes encoding antioxidant and detoxifying enzymes, such as glutathione S-transferase, glutathione synthetase, heme oxygenase 1, and NAPH-oxidoreductase.

Some enzymes involved in ROS production, such as NOS, NOX, and MPO can be found in the peripheral blood and can therefore be used as markers of oxidative stress. However, expensive equipment would be required to detect MPO, also due to its low concentration in the blood, and this severely limits its use in clinical practice. Finally, antioxidant enzymes, such as CAT and SOD, can also be used as markers of oxidative stress. Conventional methods for evaluating enzymes are: gene expression by reverse transcription polymerase chain reaction (RT-PCR), direct protein quantification by western blot or other immunological techniques (e.g., immunocytochemistry and immunohistochemistry), and evaluation of the enzymatic activity [121]. Each of the methods illustrated so far for the quantification of oxidative stress has intrinsic limitations. These limits could be overcome by simultaneously using multiple evaluation criteria for oxidative stress. A redox state index was therefore proposed, the OXY-SCORE, [122,123,124], a global index of oxidative stress, which results from the integrated evaluation of a series of pro-oxidant and anti-oxidant biomarkers.

In conclusion, beyond the specific tests and biomarkers of oxidative stress already used in clinical diagnostics, the assays currently used in experimental research could be validated, in the near future, in the clinical setting. For this to happen, the reduction of economic costs, the standardization of methodological procedures, and the overcoming of the high variability of the results are necessary, also determined in part by intra-individual differences in risk factors, pathologies, and lifestyles. High costs and poor reproducibility of the results are the two elements that currently constitute the greatest obstacles to their large-scale use in clinical diagnostics.

## 5. Oxidative Stress in Gastrointestinal Disease

The gastrointestinal tract is a key source of ROS. Many cell types within the mucosa of the GIT produce ROS as part of normal physiology, yet the gut mucosa is also a target of various oxidants that can lead to pathological conditions. Redox signaling regulates the physiological function of gastrointestinal epithelium mainly through NADPH oxidases (NOXs), and commensal bacteria also contribute to intestine epithelial homeostasis through NOX1- and DOUX2-derived ROS. Commensal gut microbiota molecules, such as N-butyrate, are essential for controlling mitochondrial oxidative stress and inflammatory responses, pathogen growth, and adherence, as well as in improving metabolism and energy expenditure during exercise [125]. Despite the protective barrier provided by the epithelial layer, ingested materials and pathogens can cause inflammation by activating the epithelium, polymorphonuclear neutrophils, and macrophages to produce inflammatory cytokines and other mediators that contribute further to oxidative stress. Moreover, an excess of ROS could also induce discontinuation of the GI tract barrier, thereby increasing intestinal permeability and contributing to the inflammation observed in a variety of gastrointestinal diseases. Among the main sources of ROS and RNS, NADPH oxidase exerts an important role in the gastrointestinal tract. This group of enzymes includes numerous membrane-bound multimeric NOX isoforms and DUOX complexes, which are present in different tracts of the gut [126]. In particular, while DUOX complexes are found in all the tracts of the intestine, NOX1 is present only in the ileum, cecum, and colon epithelium. NOX2 is expressed mainly by professional phagocytes, while NOX4 is present in the epithelium, fibroblasts, and smooth muscle cells, and its expression was induced by stimuli such as TGF-β and hypoxia [37,127,128,129]. Other sources of intestinal ROS are mitochondria, particularly electron transport chain and NO synthases (NOS) [126]. It has been demonstrated that mitochondria have a prominent role in the modulation of gut functions as intestinal barrier protection, mucosal immune response [130,131], and maintenance of an eubiotic intestinal microbiota. A crosstalk between the gut microbiota and mitochondria during exercise is known in the literature. In particular, endurance exercise induces systemic mitochondrial biogenesis, prevents mitochondrial DNA depletion and mutations, and increases mitochondrial oxidative and antioxidant capacity. However, overtraining and chronic stress promote gut inflammation in athletes, which results in a plethora of stressors that favour the lipopolysaccharide translocation and the proliferation of pathobionts [125]. Moreover, oxidative stress exerts an important role in dysbiosis through the variation of microbial diversity in the gut. Intestinal inflammation, and consequent leukocyte infiltration, trigger oxidative stress by a generation of ROS and RNS promoting the loss of anaerobic bacteria [132,133].

**Figure 4:**
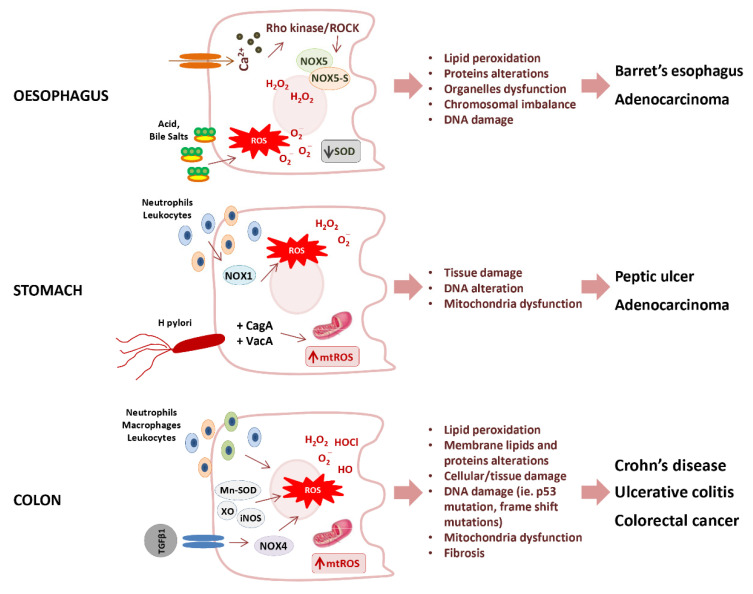
Potential mechanisms of oxidative stress promoting gastrointestinal diseases. SOD, Superoxide dismutase; ROS, reactive oxygen species; mtROS, mitochondrial reactive oxygen species; NOXs (NOX1, NOX4, NOX5, NOX5-S) NADPH oxidases; Cag A, cytotoxin-associated gene A; Vac A, vacuolating cytotoxin A; Mn-SOD, manganese-dependent superoxide dismutase; XO, xanthine oxidase; iNOS, inducible nitric oxide synthase.

The loss of redox homeostasis is implicated in the pathogenesis of several gastrointestinal disorders, such as Barrett’s esophagus, peptic ulcer, celiac disease, inflammatory bowel disease, and several adenocarcinomas (Figure 4) [134].

The Barrett’s esophagus is characterized by an increased production of O_2_^−^ anion, and consequent lipid peroxidation, paralleled by the inactivation of SOD [135]. The increase of O_2_^−^ in Barrett’s esophagus was triggered by one of the NOX isoforms. i.e., NOX5 [136], whose overexpression results mediated by the calcium-dependent activation of Rho kinase ROCK2 [137]. Moreover, it has also been observed that NOX5-S, a variant lacking calcium-binding domains, triggers the acid-induced generation of H_2_O_2_ and DNA damage in Barrett’s cells, thus contributing to the progression from Barrett’s esophagus to adenocarcinoma [138].

As previously stated, NOX1 and DUOX2 have significant roles in *Helicobacter pylori*-induced gastric inflammation, which plays a cardinal role in peptic ulcer disease and gastric cancer. In particular, peptic ulcer is characterized by overproduction of O_2_^−^ and H_2_O_2_ derived mainly from leukocyte and neutrophils infiltrate [139,140], and from NOXs activity [141]. The oncogenesis of gastric carcinoma was found associated with the upregulation of both NOX-1 and spermine oxidase. The up-regulation of spermine oxidase activity induced by *H. pylori* in gastric epithelial cells increased H_2_O_2_ as a byproduct during the conversion of polyamine spermine into spermidine with consequent oxidative DNA damage [142].

Oxidative stress was also found to mediate most of the cytotoxic effects induced by gluten peptides in intestinal epithelial cells in celiac disease. In addition, ROS also enhanced the inflammatory cascade via NF-κB, and increased transglutaminase levels [134].

Progression of inflammatory bowel disease appeared to be determined by a quite complex mechanism of balance between pro-inflammatory redox-sensitive pathways, such as NLRP3 inflammasome and NF-κB, and the adaptive up-regulation of the antioxidant enzymes Mn-SOD and glutathione peroxidase 2 (GPX2) [134].

The overproduction of ROS due to mitochondrial dysfunction plays an important role also in the pathogenesis of inflammatory bowel disease (IBD) [126]. IBD, including Crohn’s disease and ulcerative colitis, were characterized by chronic inflammation of the GI tract. In ulcerative colitis, only the colon mucosal layer is affected, whereas in Crohn’s disease, inflammation may occur in all layers of the GI tract wall. Although the exact genesis of IBD is not fully understood, the association of ROS with IBD appeared evident from the observation that increased ROS and decreased antioxidant levels represented the major pathogenetic mechanisms in IBD [63,143]. Although the two forms of IBD share similar characteristics, H_2_O_2_ and HOCl showed an important role in the pathophysiology of ulcerative colitis, whilst HO^·^ and O_2_^−^ are found to be responsible for Crohn’s disease [144]. Further, in ulcerative colitis, a loss of mucosal antioxidant defence contributes to inflammation and disease progression. Murine studies showed that the severity of ulcerative colitis is related to SOD [145] and that antioxidants significantly reduce inflammatory responses [146]. Similarly, in inflamed mucosa of Crohn’s disease patients, the increase in XO, Mn-SOD activity, iNOS, and tumor necrosis factor-α (TNF-α) resulted associated with decreased antioxidant levels [57,147]. A deficiency in antioxidant molecules could lead to increased levels of lipid peroxides or ROS, which could act locally or be secreted into the circulation to produce different systemic effects in the patient [148].

## 6. Role of Oxidative Stress in Gastrointestinal Muscular Alterations

There are numerous data on the effects of oxidative stress in skeletal muscle. For example, it has been observed that in the skeletal muscle cells, the production of ROS increased with age and was associated with a loss of function, but could also significantly increase in the case of chronic inflammation [149]. As far as smooth muscle was concerned, most of the data are related to lung and vascular diseases. In fact, many experimental data showed that vascular smooth muscle cells are involved, together with endothelial cells, in the development of atherosclerosis, a pathology characterized by persistent inflammation and oxidative stress [150].

In any case, the analysis of the experimental data highlights the role played by mitochondria in oxidative stress.

Mitochondria are involved in many cellular functions, including the production of adenosine triphosphate (ATP), redox homeostasis, ROS and NADPH generation, calcium metabolism, and apoptosis [151]. Moreover, mitochondria can also detect warning signs and induce inflammation by activating and controlling the innate immune system [152]. Given the importance of mitochondria, alterations in their functions can have a profound effect on immunology and cell biology.

For instance, abnormalities in mitochondrial function have been described in human airway smooth muscle (ASM) cells from asthmatic patients [153], and in bronchial epithelial cells from ex-smokers with chronic obstructive pulmonary disease (COPD) [154]. In both pathologies, there are excessive mitochondrial ROS production, damaged mitochondrial structures with depletion of cristae, increased branching, elongation, and swelling of the mitochondria. Moreover, ASM cells from severe asthmatic patients present also a lack in the NRF2 antioxidant system [153].

Furthermore, it has been observed that oxidative damage can cause lesions of endothelial cells and deleterious vasodilatory effects, which could induce functional alterations in the smooth muscle cells of the vessel wall [155,156].

As regards the pathologies of the gastrointestinal tract, while the mucosal alterations associated with oxidative stress have been extensively investigated, the knowledge on oxidative stress-mediated muscle alterations has been only recently expanding. Most of the literature data come from studies on animal models, and the role of oxidative stress in the pathogenesis of muscular gastrointestinal diseases has not yet been extensively studied in humans.

Past studies performed on the murine model showed that molecules involved in oxygen-free radical production or in protection against oxygen radicals differed among the different gastrointestinal tracts and suggested that the large intestine was better provided with protective enzymes and non-enzymatic factors against oxidative stress than the small intestine [39]. Accordingly, it was observed that the large intestine was the most sensitive gastrointestinal tract in which oxidative stress-induced an alteration of intestinal motility. In fact, treatment of tissue segments from the large intestine with hydrogen peroxide compromised the contractile response into muscarinic agonists [39].

Later, Gonzalez and co-workers studied the in vitro modulation of rat colonic circular muscle contractions by dextran sodium sulfate (DSS)-induced inflammation coming to the same conclusions. They observed that H_2_O_2_ altered the excitation-contraction coupling ending process suppressing the spontaneous phasic contractions and reducing responses by acetylcholine (ACh) stimuli [157]. The suppression of contractile capacity induced by oxidative stress was also confirmed by a study conducted on normal and inflamed canine colon, which demonstrated as H_2_O_2_-induced oxidative stress activated NF-κB in colonic circular smooth muscle cells, resulting in suppression of their contractility [158].

Similarly, it has been reported that in the intestines of rats, oxidative stress associated with aging reduced the tone of the internal anal sphincter (IAS) via RhoA/ROCK down-regulation. In particular, the decrease of RhoA/ROCK expression, both at the transcriptional and translational levels, was reverted by the activity of SOD, thus demonstrating its link with oxidative stress [159]. However, these data disagree with a previous observation made on rat gastric muscle in which oxidative stress induced an activation of Rho kinase II with a consequent increase in Ach-induced contraction [160]. Nevertheless, in a later study, Singh and co-authors described a bimodal effect of oxidative stress in IAS basal tone. Mild oxidative stress led to an increase in IAS tone associated, at least in part, with neuronal nitric oxide synthase (nNOS) inhibition; on the other hand, higher levels of oxidative stress caused a decrease in IAS tone. Both these effects were associated with changes in RhoA/ROCK [159]. The bimodal effect of oxidative stress was being previously hypothesized.

H_2_O_2_ has been shown to have not only harmful effects, since it results in an important signaling molecule that stimulates cell growth/proliferation and DNA synthesis in different types of cells [161,162]. More recently, Song et al. showed in feline ileal smooth muscle cells as short-term oxidative stress induced by H_2_O_2_ activated the signal transduction of mitogenic pathways, which are thought to represent a protective response against oxidant injury [163].

Only recently, the effects of oxidative stress on smooth muscle have been analyzed in human gastrointestinal muscle tissues. Scirocco et al. provided the first direct evidence of a muscular oxidative imbalance in the human gastric muscle that impaired antral smooth muscle relaxation both in vivo and in vitro [164]. This observation was in accordance with previous data showing the association between metabolic disorders and oxidative stress [165], and its effects on vascular and cardiac smooth muscle relaxation in the obesity condition [166]. In gastric muscle, the oxidative imbalance mainly affects the cAMP-signaling pathway and the expression of eNOS, two key components of the vasoactive intestinal polypeptide (VIP)-induced relaxation. The key role of oxidative stress in the impairment of muscle activity was confirmed by the ability of apocynin, an NADPH-inhibitor, to restore relaxation as well as antioxidant cell capacity and eNOS expression [164].

Interestingly, oxidative-stress mediated muscle impairment was also observed in vitro when the colonic muscle was exposed to supernatants obtained from a culture of mucosal biopsies of patients with irritable bowel syndrome (IBS). Colonic human smooth muscle strips and cells showed a decreased basal tone, a significant cell shortening, and a reduced Ach-induced contraction after exposure to supernatants [167]. The oxidative-related muscle damage induced by mucosal supernatants occurred likely through the generation of superoxide rather than hydrogen peroxide damage since it was reverted by apocynin but not by catalase [167].

Studies on Crohn’s disease have shown an increase in ROS levels and a decrease in antioxidant defenses resulting in a state of oxidative stress at the level of stenosis tracts [168]. Intestinal fibrosis with stricture formation represents a severe complication in Crohn’s disease. It has recently been reported that fibrosis could be triggered by NOX4-dependent ROS production that, activating myofibroblasts cells to produce collagen, lead to an increased thickness of the bowel wall [148,169].

**Figure 5:**
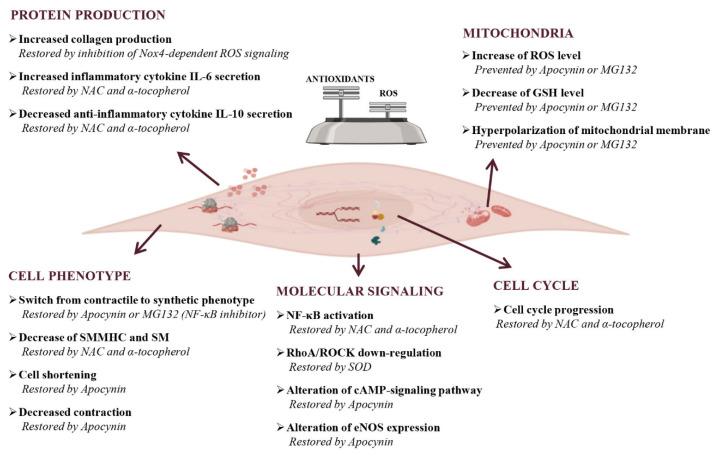
Effects of oxidative stress on gastrointestinal smooth muscle cells. Oxidative stress can cause both cell structure-function alterations and inflammation with the promotion of pro-inflammatory environments. As concern cell phenotype, ROS alter the production of cytoskeletal proteins, like smooth muscle myosin heavy chain (SMMHC) and smoothelin (SM) that leads to the impairment of contraction and cell length. The final consequence of these alterations is the cellular switch from contractile to the synthetic phenotype. In the presence of oxidative stress, molecular signaling results are also altered. In particular, signaling that leads to the amplification of damage (NF-κB-signaling) are activated, complexes involved in the correct maintenance of contractile phenotype (RhoA/ROCK-signaling) are down regulated, and eNOS expression and cAMP-signaling pathways resulted are altered. ROS have a dual effect on cells: they induce apoptosis but also favour the cell cycle progression as compensatory mechanism. An increase of ROS, or conversely a decrease of antioxidants, induce cells to an oxidative state that manifests itself also with hyperpolarization of mitochondrial membrane. The last effect of oxidative stress on cells is the alterations of protein production, in particular extracellular proteins, like collagen, and pro-inflammatory cytokines IL-6 result increased, while anti-inflammatory cytokines IL-10 result decreased. The presence of an oxidative status is confirmed by the reversion or prevention of cellular oxidative damages after antioxidants and/or inhibitor of molecular patterns treatment. NAC, N-acetylcysteine, Nox, NADPH oxidases; SOD, superoxide dismutase.

The role of free radicals, lipid peroxides, and antioxidant activities in the occurrence of muscular phenotypic switch remains to be clarified. An oxidative imbalance-driven phenotypic switch of human colonic smooth muscle cells (HSMC) has been demonstrated in response to bacterial lipopolysaccharide (LPS) exposure via its interaction with toll-like receptor 4 (TLR4), constitutively expressed by colonic SMC [170]. LPS-induced contractile dysfunction in SMC, consisting of a time- and dose-dependent decrease in cell length and contraction, was associated with ROS production, GSH content depletion, hyperpolarization of mitochondrial membrane, and rearrangement of actin microfilament cytoskeleton. Most of these effects were partially prevented by the NADPH oxidase inhibitor apocynin or the NF-κB inhibitor MG132, supporting the important pathogenic role of oxidative stress (Figure 5) [171]. Thus, the exposure to bacterial endotoxin directly and persistently impaired gastrointestinal smooth muscle activity, strongly indicating that HSMC could actively participate in the dysmotility observed during the infective burst.

In addition, prolonged exposure to LPS also triggers a redox imbalance that leads to profound modifications of the contractile microfilament network, with a decrease of the contractile differentiation markers smooth muscle myosin heavy chain and smoothelin, and the induction of cell proliferation, thus inducing a persistent cellular phenotype switch from a contractile to a synthetic phenotype [172]. These effects were potently counteracted by antioxidant drugs alpha-tocopherol and N-acetylcysteine (NAC), which were able to reverse the cytopathic effects of LPS and to restore normal muscle cell function.

Very interestingly, oxidative damage has also been reported on vascular smooth muscle cells (VSMCs), major cell types in the vessel wall, which has different functions. As colonic SMC, VSMCs present two different phenotypes: contractile and synthetic [173]. When subject to inflammation and oxidative stress, VSMCs switch from the contractile to the synthetic phenotype. It was hypothesized that the phenotypic switch may play an important role in several cardiovascular diseases [174]. Indeed, the phenotypic switch, while causing the loss of the contractile ability of the VSMC, gives it the ability to recruit inflammatory cells, thus inducing a remodeling of the vessel wall at the basis of the vascular damage [173]. The crosstalk between oxidative stress and inflammation therefore represents a key element in endothelial dysfunction and consequent vascular damage. Endothelial dysfunction is known to be characterized by an impaired ability to regulate vascular tone and altered anti-inflammatory and anticoagulant properties [155,174]. For this reason, endothelial dysfunction has been accepted as primum movens in the development of hypertension [175] and atherosclerosis [156,176].

By contrast, the exact molecular mechanisms underlying the intestinal muscle alterations induced by oxidative stress remain to be clarified. This would be very important in order to provide new insights in the pathophysiology of persistent gut dysmotility disorders that occur, for instance, in post-infective conditions (i.e., post-infective IBS) [177] or during remission of IBD.

Furthermore, shedding light on the role of oxidative stress in gut dysmotility disorders is essential for the development of new therapeutic approaches, also with a possible reappraisal of antioxidants molecules in the clinical management of these disorders.

## 7. Therapeutic Strategies Based on the Use of Antioxidants

Currently, the treatments of gastrointestinal inflammatory diseases, such as CD and UC, include a combination of immunosuppressive agents and anti-inflammatory, although sometimes adverse effects may occur [178,179]. The treatment can also include probiotics and prebiotics in order to normalize the microbiota favoring the species with a greater antioxidant capacity [180]. Importantly, modifications in diet and lifestyle are always recommended. Pharmacological treatments aim at blocking TNF-α or NF-κB-mediated inflammation. Given the close connection between inflammation and oxidative stress, the use of antioxidant supplements could modulate endogenous mechanisms of ROS production, through inhibition of oxidizing enzymes, or enhancing the antioxidant system activity (Table 4). As mentioned above, the role of oxidative stress, either as the main cause or secondary effect of the inflammation, was been demonstrated in inflammatory bowel disease, gastritis, and peptic ulcer disease and to a lesser extent, in celiac disease, inflammatory bowel syndrome, and esophagus cancer. Hence, modulation of oxidative stress represents an evidence-based rational choice to improve gastrointestinal disorders.

Among the antioxidants, polyphenols are widely used in the treatment of gastrointestinal diseases. In particular, they have antioxidant, anti-inflammatory, and immunomodulatory action. Polyphenols are able to inhibit the cytokines production, such as IL-8, IL-1β, and TNF-α, to promote the activities of intracellular antioxidants, including SOD and GPX, and to directly scavenge free radicals. In addition, polyphenols protect the intestinal mucosal by reducing intestinal permeability via tight junction stabilization. They also enhance the healthy microbiota in the gut [181]. Polyphenols include flavonoids and phenolic acids (Figure 3). Among the polyphenols, resveratrol is used in the treatment of *H. Pylori*- related disease [182] and to improve IBD [183,184,185], thanks to its anti-inflammatory and antioxidant properties. Moreover, this compound has also been reported to inhibit the proliferation of gastric cancer cells [186].

Studies in animal models have also shown that antioxidants such as curcumin, boswellic acids, planar aromatics, and rosmarinic acid were able to activate the NRF2 pathway [187,188,189,190,191]. The antioxidant supplements may also decrease ROS levels by affecting specific enzymes. For example, allopurinol inhibited the action of xanthine oxidase that generates O_2_^•^^−^. Curcumin hindered inflammation by decreasing COX activities, IL-1β, and the AKT/mTOR pathway. Several clinical studies have reported the benefits of curcumin in IBD patients without serious side-effects [192,193]. Flavonoids prevent ROS production by inhibiting XO [194], COX, LOX, GST, and NADH oxidase [195]. Moreover, several flavonoids are also able to chelate free Fe^++^ and Cu^++^ that could increase ROS generation [196].

Recently, new technologies and nanomaterials have been developed that can improve the targeted administration of antioxidant drugs in gastrointestinal inflammation [197,198]. In particular, nanomaterials can be designed in such a way as to have antioxidant properties themselves, thus acting as nano-antioxidants, or as carriers and/or containers of natural compounds with antioxidant activity, mainly polyphenols [199]. Many literature data have reported the use of exosomes, lipid, and magnetic polymers; natural and synthetic polymers; ultrasonic microtubules; and nanoemulsions for the targeted release of antioxidants, in particular curcumin [200,201,202], resveratrol, and vitamin E [203] in the treatment of gastrointestinal inflammation, mostly IBD [204] and gastric ulceration [205]. It is very important to note that, although the use of nanostructures is associated with better bioavailability or enhancing pharmacokinetics, at high concentrations, they could be very toxic, releasing the drug out of control, and increasing oxidative stress through the generation of ROS. Therefore, they need further studies before any preclinical and clinical applications [199].

## 8. Conclusions

Generally, our body counterbalances the production of reactive species with the endogenous antioxidant defense system or by antioxidants introduced with the diet. When this balance is not maintained, oxidative stress occurs. Oxidative stress contributes to the evolution of numerous and diverse pathologies also those of the gastrointestinal tract. As shred of evidence shows that the use of antioxidants can improve the evolution of many diseases, the development of antioxidant therapies represents a promising avenue also for the gastrointestinal pathologies treatment. Therefore, the knowledge of the peculiar oxidative pathway involved in each disease could allow both the identification of disease markers and the development of preventive and curative therapeutic strategies.

## Figures and Tables

**Table 1:** Fluorescent probes used for the measurements of reactive oxygen and nitrogen species by flow cytometry.

Probe (Localization)	ROS/RNS	Limitations
DCFH-DA (intracellular)	^•^OH, ONOO^−^, ^•^NO_2_, H_2_O_2_	DCF radicals production, MDR substrates or inducers, Antioxidants
DAF-2 DA/DAF-FMDA(intracellular)	^•^NO	MDR substrates or inducers, Esterase inhibitors
DHR123 (intracellular)	H_2_O_2_	MDR substrates or inducers, DHR radicals production
HE (intracellular)	O_2_^•−^	Intercalating agents
C11-BODIPY^581/591^ (membrane)	^•^OH, ^•^ROO	Antioxidants

C11-BODIPY581/591: 4,4-difluoro-5-(4-phenyl-1,3-butadienyl)-4-bora-3a,4a-diaza-s-indacene-3-undecanoic acid; DAF-2 DA: 4,5-diaminofluorescein diacetate; DAF-FMDA: 4-amino-5-methylamino-2′,7′-difluorofluorescein diacetate; DCFH-DA: dihydrochlorofluorescein diacetate; DHR123: dihydrorhodamine 123; H_2_O_2_: hydrogen peroxide; HE: hydroethidine; MDR: multidrug resistance; ^•^NO: nitrogen monoxide; NO_2_^•^: nitrogen dioxide; O_2_^•−^: superoxide radical; ^•^HO: hydroxyl radical; ONOO^−^: peroxynitrite; ^•^ROO: peroxyl radicals.

**Table 2:** Markers obtained from ROS-induced modifications.

Markers	Methods	Limitations
Lipid oxidation		
HNE	HPLC, GC-MS, Immunoassay	Sugars, aminoacids, Bilirubin and albumin, antibody specificity
MDA	Spectrophotometric/fluorimetric (TBARS), HPLC, Immunoassay	
F2-IsoPs	Gas/liquid chromatography, Immunoassay	Antibody specificity
DNA oxidation		
8oxodG 5-chlorocytosine 5-chlorouracil	ELISA assays, HPLC-ECD, HPLC/GC-MS, Western blot, immunohistochemistry	Antibody specificity
Protein oxidation		
ALEs, AGEs	HPLC, Western blot, immunohistochemistry, ELISA	Structural variety of products, Antibody specificity
Carbonils	Spectrophotometric, HPLC, ELISA, Western blot	
3-NO-Tyr	HPLC/GC-MS, ELISA, Flow cytometry	Possible nitration of tyrosine residues in the sample, Antibody specificity
AOPP	MS, colorimetric assays	
oxLDL	ELISA, Flow cytometry	Antibody specificity
IMA	ABC test, ELISA	Antibody specificity

8oxodG: 7,8-dihydroxy-8-oxo-2′-deoxyguanosine; ABC test: binding capacity of albumin for cobalt; AGEs: advanced glycation end products; ALEs: advanced lipoxigenation end products; AOPP: advanced oxidation protein products; F2-IsoPs: F2-isoprostanes; GC: gas chromatography; HNE: 4-hydroxy-2-nonenal; HPLC: high-performance liquid chromatography; IMA: ischemia-modified albumin; MS: mass spectroscopy; MDA: malondialdehyde; TBARS: thiobarbituric acid reactive substances.

**Table 3:** Markers of antioxidant defenses.

Markers	Methods	Limitations
Cysteine modifications		
S-glutathioylation	MS, ELISA, WB	Specialized instrumentation
GSH/GSSG, SH	Spectrophotometric, Flow cytometry	
ROS-regulated transcription factors		
Nrf2, NF-kB	RT-PCR, WB, immunohistochemistry	Antibody specificity
ROS-generating enzyme		
NOX, MPO, XO, NOS	RT-PCR, WB, ELISA, Immunological techniques	Antibody specificity
Antioxidant enzymes		
SOD, CAT, GPX, GR	ELISA, WB, PCR, RT-PCR, Immunological techniques	Antibody specificity

CAT: catalase; GPX: glutathione peroxidase; GR: glutathione reductase; GSH: glutathione; MPO: Myeloperoxidase; MS: mass spectroscopy; NOS: nitric oxide synthases; NOX: NADPH oxidase; PCR: reverse-transcription polymerase chain reaction; RT-PCR: reverse-transcription polymerase chain reaction; SOD: superoxide dismutase; WB: Western blot; XO: xanthine oxidase.

**Table 4:** Therapeutic antioxidant strategies.

Compounds	Effects	GI Disease	Ref.
Melatonin	Anti-inflammatory and anti-oxidant action Lipid peroxidation reduction MAPK and NF-kB Activation iNOS expression induction Stimulation of nitrite production in intestinal tissue	PI-IBS	[185]
Polyphenols	Inhibition of cytokines production Promotion of intracellular antioxidants activity Scavenging direct of free radicals Protection of intestinal mucosal Enhancing of eubiosis	IBD	[181]
SPECIFIC POLYPHENOLS			
Resveratrol	Anti-inflammatory and anti-oxidant activity Inhibition of gastric cancer cells proliferation	IBD *H. pylor*i-related disease	[182,183,186]
Allopurinol	Inhibition of XO activity	ID	[192,193]
Curcumin	Inhibition of COX activity Inhibition of IL-1β Inhibition of AKT/mTOR pathway	IBD	[192,193]
Flavonoids	Inhibition of XO, COX, LOX, GST, and NOX Chelation of free Fe^++^ and Cu^++^	IBD	[184,194,195,196]

PI-IBS: post-infective Irritable Bowel Syndrome; IBD: Inflammatory Bowel Disease; ID: Inflammatory Bowel Disease; NOX: NADPH oxidase; XO: xanthine oxidase; COX: cyclooxygenases; LOXs: lipoxygenases; GST: glutathione transferase; iNOS: inducible nitric oxide synthase.

## References

[1] Janssen-Heininger Y.M., Mossman B.T., Heintz N.H., Forman H.J., Kalyanaraman B., Finkel T., Stamler J.S., Rhee S.G., van der Vliet A. (2008). Redox-based regulation of signal transduction: Principles, pitfalls, and promises. Free Radic. Biol. Med..

[2] Vaziri N.D. (2008). Causal link between oxidative stress, inflammation, and hypertension. Iran. J. Kidney Dis..

[3] Petrie J.R., Guzik T.J., Touyz R.M. (2018). Diabetes, Hypertension, and Cardiovascular Disease: Clinical Insights and Vascular Mechanisms. Can. J. Cardiol..

[4] Ishibashi T. (2013). Molecular hydrogen: New antioxidant and anti-inflammatory therapy for rheumatoid arthritis and related diseases. Curr. Pharm. Des..

[5] Ng C.Y., Kamisah Y., Faizah O., Jaarin K. (2012). The role of repeatedly heated soybean oil in the development of hypertension in rats: Association with vascular inflammation. Int. J. Exp. Pathol..

[6] Vona R., Gambardella L., Cittadini C., Straface E., Pietraforte D. (2019). Biomarkers of Oxidative Stress in Metabolic Syndrome and Associated Diseases. Oxid. Med. Cell Longev..

[7] Violi F., Loffredo L., Carnevale R., Pignatelli P., Pastori D. (2017). Atherothrombosis and Oxidative Stress: Mechanisms and Management in Elderly. Antioxid. Redox Signal..

[8] Cai H., Harrison D.G. (2000). Endothelial dysfunction in cardiovascular diseases: The role of oxidant stress. Circ. Res..

[9] Majzunova M., Dovinova I., Barancik M., Chan J.Y. (2013). Redox signaling in pathophysiology of hypertension. J. Biomed. Sci..

[10] Klaunig J.E. (2018). Oxidative Stress and Cancer. Curr. Pharm. Des..

[11] Reuter S., Gupta S.C., Chaturvedi M.M., Aggarwal B.B. (2010). Oxidative stress, inflammation, and cancer: How are they linked?. Free Radic. Biol. Med..

[12] Islam M.T. (2017). Oxidative stress and mitochondrial dysfunction-linked neurodegenerative disorders. Neurol. Res..

[13] Singh A., Kukreti R., Saso L., Kukreti S. (2019). Oxidative Stress: A Key Modulator in Neurodegenerative Diseases. Molecules.

[14] Wang J., Wang H. (2017). Oxidative Stress in Pancreatic Beta Cell Regeneration. Oxid. Med. Cell Longev..

[15] Noh S., Go A., Kim D.B., Park M., Jeon H.W., Kim B. (2020). Role of Antioxidant Natural Products in Management of Infertility: A Review of Their Medicinal Potential. Antioxidants (Basel).

[16] Ratliff B.B., Abdulmahdi W., Pawar R., Wolin M.S. (2016). Oxidant Mechanisms in Renal Injury and Disease. Antioxid. Redox Signal..

[17] Puentes-Pardo J.D., Moreno-SanJuan S., Carazo A., Leon J. (2020). Heme Oxygenase-1 in Gastrointestinal Tract Health and Disease. Antioxidants (Basel).

[18] Kekec Y., Paydas S., Tuli A., Zorludemir S., Sakman G., Seydaoglu G. (2009). Antioxidant enzyme levels in cases with gastrointesinal cancer. Eur. J. Intern. Med..

[19] Inokuma T., Haraguchi M., Fujita F., Tajima Y., Kanematsu T. (2009). Oxidative stress and tumor progression in colorectal cancer. Hepatogastroenterology.

[20] Zhang L., Li L., Gao G., Wei G., Zheng Y., Wang C., Gao N., Zhao Y., Deng J., Chen H. (2017). Elevation of GPRC5A expression in colorectal cancer promotes tumor progression through VNN-1 induced oxidative stress. Int. J. Cancer.

[21] Grisham M.B. (1994). Oxidants and free radicals in inflammatory bowel disease. Lancet.

[22] Pavlick K.P., Laroux F.S., Fuseler J., Wolf R.E., Gray L., Hoffman J., Grisham M.B. (2002). Role of reactive metabolites of oxygen and nitrogen in inflammatory bowel disease. Free Radic. Biol. Med..

[23] Peng Y.C., Hsu C.L., Tung C.F., Chou W.K., Huang L.R., Hung D.Z., Hu W.H., Yang D.Y. (2008). Chemiluminescence assay of mucosal reactive oxygen species in gastric cancer, ulcer and antral mucosa. Hepatogastroenterology.

[24] Bhattacharyya A., Chattopadhyay R., Mitra S., Crowe S.E. (2014). Oxidative stress: An essential factor in the pathogenesis of gastrointestinal mucosal diseases. Physiol. Rev..

[25] Pham-Huy L.A., He H., Pham-Huy C. (2008). Free radicals, antioxidants in disease and health. Int. J. Biomed. Sci..

[26] Cheeseman K.H., Slater T.F. (1993). An introduction to free radical biochemistry. Br. Med. Bull..

[27] Lobo V., Patil A., Phatak A., Chandra N. (2010). Free radicals, antioxidants and functional foods: Impact on human health. Pharm. Rev..

[28] Davies M.J., Fu S., Wang H., Dean R.T. (1999). Stable markers of oxidant damage to proteins and their application in the study of human disease. Free Radic. Biol. Med..

[29] Dalle-Donne I., Scaloni A., Giustarini D., Cavarra E., Tell G., Lungarella G., Colombo R., Rossi R., Milzani A. (2005). Proteins as biomarkers of oxidative/nitrosative stress in diseases: The contribution of redox proteomics. Mass Spectrom. Rev..

[30] Balaban R.S., Nemoto S., Finkel T. (2005). Mitochondria, oxidants, and aging. Cell.

[31] Schieber M., Chandel N.S. (2014). ROS function in redox signaling and oxidative stress. Curr. Biol..

[32] Ray P.D., Huang B.W., Tsuji Y. (2012). Reactive oxygen species (ROS) homeostasis and redox regulation in cellular signaling. Cell Signal..

[33] Giles G.I., Nasim M.J., Ali W., Jacob C. (2017). The Reactive Sulfur Species Concept: 15 Years On. Antioxidants (Basel).

[34] Ali S.S., Ahsan H., Zia M.K., Siddiqui T., Khan F.H. (2020). Understanding oxidants and antioxidants: Classical team with new players. J. Food Biochem..

[35] Munzel T., Gori T., Bruno R.M., Taddei S. (2010). Is oxidative stress a therapeutic target in cardiovascular disease?. Eur. Heart J..

[36] Swindle E.J., Metcalfe D.D. (2007). The role of reactive oxygen species and nitric oxide in mast cell-dependent inflammatory processes. Immunol. Rev..

[37] Bedard K., Krause K.H. (2007). The NOX family of ROS-generating NADPH oxidases: Physiology and pathophysiology. Physiol. Rev..

[38] O’Neill S., Brault J., Stasia M.-J., Knaus U.G. (2015). Genetic disorders coupled to ROS deficiency. Redox Biol..

[39] Van der Vliet A., Tuinstra T.J., Bast A. (1989). Modulation of oxidative stress in the gastrointestinal tract and effect on rat intestinal motility. Biochem. Pharm..

[40] Zhu H., Yang L., Zhou B., Yu R., Tang N., Wang B. (2006). Myeloperoxidase G-463A polymorphism and the risk of gastric cancer: A case-control study. Carcinogenesis.

[41] Ullman T.A., Itzkowitz S.H. (2011). Intestinal inflammation and cancer. Gastroenterology.

[42] Peek R.M., Fiske C., Wilson K.T. (2010). Role of innate immunity in Helicobacter pylori-induced gastric malignancy. Physiol. Rev..

[43] Barrachina M.D., Panes J., Esplugues J.V. (2001). Role of nitric oxide in gastrointestinal inflammatory and ulcerative diseases: Perspective for drugs development. Curr. Pharm. Des..

[44] Guerra D.D., Bok R., Vyas V., Orlicky D.J., Lorca R.A., Hurt K.J. (2019). Akt phosphorylation of neuronal nitric oxide synthase regulates gastrointestinal motility in mouse ileum. Proc. Natl. Acad. Sci. USA.

[45] Marnett L.J. (2009). The COXIB experience: A look in the rearview mirror. Annu. Rev. Pharm. Toxicol..

[46] Mahkonen A., Putaala H., Mustonen H., Rautonen N., Puolakkainen P. (2008). Lactobacillus acidophilus 74-2 and butyrate induce cyclooxygenase (COX)-1 expression in gastric cancer cells. Immunopharmacol. Immunotoxicol..

[47] Lassegue B., Sorescu D., Szocs K., Yin Q., Akers M., Zhang Y., Grant S.L., Lambeth J.D., Griendling K.K. (2001). Novel gp91(phox) homologues in vascular smooth muscle cells: Nox1 mediates angiotensin II-induced superoxide formation and redox-sensitive signaling pathways. Circ. Res..

[48] Brigelius-Flohe R., Flohe L. (2011). Basic principles and emerging concepts in the redox control of transcription factors. Antioxid. Redox Signal..

[49] Surh Y.J., Kundu J.K., Li M.H., Na H.K., Cha Y.N. (2009). Role of Nrf2-mediated heme oxygenase-1 upregulation in adaptive survival response to nitrosative stress. Arch. Pharm. Res..

[50] Halliwell B. (1995). How to characterize an antioxidant: An update. Biochem. Soc. Symp..

[51] Halliwell B., Gutteridge J.M. (1995). The definition and measurement of antioxidants in biological systems. Free Radic. Biol. Med..

[52] Halliwell B., Aeschbach R., Loliger J., Aruoma O.I. (1995). The characterization of antioxidants. Food Chem. Toxicol..

[53] Rahman K. (2007). Studies on free radicals, antioxidants, and co-factors. Clin. Interv. Aging.

[54] Nozik-Grayck E., Suliman H.B., Piantadosi C.A. (2005). Extracellular superoxide dismutase. Int. J. Biochem. Cell Biol..

[55] Okado-Matsumoto A., Fridovich I. (2001). Subcellular distribution of superoxide dismutases (SOD) in rat liver: Cu,Zn-SOD in mitochondria. J. Biol. Chem..

[56] Poyton R.O., Ball K.A., Castello P.R. (2009). Mitochondrial generation of free radicals and hypoxic signaling. Trends Endocrinol. Metab..

[57] Kruidenier L., Kuiper I., van Duijn W., Marklund S.L., van Hogezand R.A., Lamers C.B., Verspaget H.W. (2003). Differential mucosal expression of three superoxide dismutase isoforms in inflammatory bowel disease. J. Pathol..

[58] Naito Y., Yoshikawa T., Ando T., Kishi A., Ueda S., Oyamada H., Kondo M. (1992). Changes in superoxide dismutase activity in the gastric mucosa of peptic ulcer patients. J. Clin. Gastroenterol..

[59] Janssen A.M., Bosman C.B., van Duijn W., Oostendorp-van de Ruit M.M., Kubben F.J., Griffioen G., Lamers C.B., van Krieken J.H., van de Velde C.J., Verspaget H.W. (2000). Superoxide dismutases in gastric and esophageal cancer and the prognostic impact in gastric cancer. Clin. Cancer Res..

[60] Klinowski E., Broide E., Varsano R., Eshchar J., Scapa E. (1996). Superoxide dismutase activity in duodenal ulcer patients. Eur. J. Gastroenterol. Hepatol..

[61] Schrader M., Fahimi H.D. (2006). Peroxisomes and oxidative stress. Biochim. Biophys. Acta.

[62] Chang D., Hu Z.L., Zhang L., Zhao Y.S., Meng Q.H., Guan Q.B., Zhou J., Pan H.Z. (2012). Association of catalase genotype with oxidative stress in the predication of colorectal cancer: Modification by epidemiological factors. Biomed. Environ. Sci..

[63] Iborra M., Moret I., Rausell F., Bastida G., Aguas M., Cerrillo E., Nos P., Beltran B. (2011). Role of oxidative stress and antioxidant enzymes in Crohn’s disease. Biochem. Soc. Trans..

[64] Chang J.C., van der Hoeven L.H., Haddox C.H. (1978). Glutathione reductase in the red blood cells. Ann. Clin. Lab. Sci..

[65] Toppo S., Vanin S., Bosello V., Tosatto S.C. (2008). Evolutionary and structural insights into the multifaceted glutathione peroxidase (Gpx) superfamily. Antioxid. Redox Signal..

[66] Wingler K., Muller C., Schmehl K., Florian S., Brigelius-Flohe R. (2000). Gastrointestinal glutathione peroxidase prevents transport of lipid hydroperoxides in CaCo-2 cells. Gastroenterology.

[67] Chu F.F., Esworthy R.S., Doroshow J.H. (2004). Role of Se-dependent glutathione peroxidases in gastrointestinal inflammation and cancer. Free Radic. Biol. Med..

[68] Thomas D., Cherest H., Surdin-Kerjan Y. (1991). Identification of the structural gene for glucose-6-phosphate dehydrogenase in yeast. Inactivation leads to a nutritional requirement for organic sulfur. EMBO J..

[69] Arner E.S. (2009). Focus on mammalian thioredoxin reductases--important selenoproteins with versatile functions. Biochim. Biophys. Acta.

[70] Lechner S., Muller-Ladner U., Schlottmann K., Jung B., McClelland M., Ruschoff J., Welsh J., Scholmerich J., Kullmann F. (2002). Bile acids mimic oxidative stress induced upregulation of thioredoxin reductase in colon cancer cell lines. Carcinogenesis.

[71] Koharyova M., Kolarova M. (2008). Oxidative stress and thioredoxin system. Gen. Physiol. Biophys..

[72] Wang Y., Hekimi S. (2016). Understanding Ubiquinone. Trends Cell Biol..

[73] Forsmark-Andree P., Dallner G., Ernster L. (1995). Endogenous ubiquinol prevents protein modification accompanying lipid peroxidation in beef heart submitochondrial particles. Free Radic. Biol. Med..

[74] Forsmark-Andree P., Ernster L. (1994). Evidence for a protective effect of endogenous ubiquinol against oxidative damage to mitochondrial protein and DNA during lipid peroxidation. Mol. Asp. Med..

[75] Sarmiento A., Diaz-Castro J., Pulido-Moran M., Kajarabille N., Guisado R., Ochoa J.J. (2016). Coenzyme Q10 Supplementation and Exercise in Healthy Humans: A Systematic Review. Curr. Drug Metab..

[76] Bentinger M., Brismar K., Dallner G. (2007). The antioxidant role of coenzyme Q. Mitochondrion.

[77] Zhang D.D., Hannink M. (2003). Distinct cysteine residues in Keap1 are required for Keap1-dependent ubiquitination of Nrf2 and for stabilization of Nrf2 by chemopreventive agents and oxidative stress. Mol. Cell Biol..

[78] Mann G.E., Niehueser-Saran J., Watson A., Gao L., Ishii T., de Winter P., Siow R.C. (2007). Nrf2/ARE regulated antioxidant gene expression in endothelial and smooth muscle cells in oxidative stress: Implications for atherosclerosis and preeclampsia. Sheng Li Xue Bao.

[79] Berger M.M. (2005). Can oxidative damage be treated nutritionally?. Clin. Nutr..

[80] Sies H. (2007). Total antioxidant capacity: Appraisal of a concept. J. Nutr..

[81] Vertuani S., Angusti A., Manfredini S. (2004). The antioxidants and pro-antioxidants network: An overview. Curr. Pharm. Des..

[82] Gale C.R. (2001). Dietary antioxidants and dementia. Int. Psychogeriatr..

[83] Block G., Patterson B., Subar A. (1992). Fruit, vegetables, and cancer prevention: A review of the epidemiological evidence. Nutr. Cancer.

[84] Yamada T., Hayasaka S., Shibata Y., Ojima T., Saegusa T., Gotoh T., Ishikawa S., Nakamura Y., Kayaba K., Jichi Medical School Cohort Study (2011). Frequency of citrus fruit intake is associated with the incidence of cardiovascular disease: The Jichi Medical School cohort study. J. Epidemiol..

[85] Mursu J., Virtanen J.K., Tuomainen T.P., Nurmi T., Voutilainen S. (2014). Intake of fruit, berries, and vegetables and risk of type 2 diabetes in Finnish men: The Kuopio Ischaemic Heart Disease Risk Factor Study. Am. J. Clin. Nutr..

[86] Kruk J. (2014). Association between vegetable, fruit and carbohydrate intake and breast cancer risk in relation to physical activity. Asian Pac. J. Cancer Prev..

[87] Kyro C., Skeie G., Loft S., Landberg R., Christensen J., Lund E., Nilsson L.M., Palmqvist R., Tjonneland A., Olsen A. (2013). Intake of whole grains from different cereal and food sources and incidence of colorectal cancer in the Scandinavian HELGA cohort. Cancer Causes Control..

[88] Azlina M.F.N., Qodriyah M.S., Kamisah Y. (2018). Tocopherol and Tocotrienol: Therapeutic Potential in Animal Models of Stress. Curr. Drug Targets.

[89] Reiter E., Jiang Q., Christen S. (2007). Anti-inflammatory properties of alpha- and gamma-tocopherol. Mol. Asp. Med..

[90] Singh M., Suman S., Shukla Y. (2014). New Enlightenment of Skin Cancer Chemoprevention through Phytochemicals: In Vitro and In Vivo Studies and the Underlying Mechanisms. Biomed. Res. Int..

[91] Koren E., Kohen R., Ginsburg I. (2010). Polyphenols enhance total oxidant-scavenging capacities of human blood by binding to red blood cells. Exp. Biol. Med. (Maywood).

[92] Zhang Y.J., Gan R.Y., Li S., Zhou Y., Li A.N., Xu D.P., Li H.B. (2015). Antioxidant Phytochemicals for the Prevention and Treatment of Chronic Diseases. Molecules.

[93] Hutchins-Wolfbrandt A., Mistry A.M. (2011). Dietary turmeric potentially reduces the risk of cancer. Asian Pac. J. Cancer Prev..

[94] Dominguez-Avila J.A., Villa-Rodriguez J.A., Montiel-Herrera M., Pacheco-Ordaz R., Roopchand D.E., Venema K., Gonzalez-Aguilar G.A. (2020). Phenolic Compounds Promote Diversity of Gut Microbiota and Maintain Colonic Health. Dig. Dis. Sci..

[95] World Health Organization, International Programme on Chemical Safety (2001). Biomarkers in Risk Assessment: Validity and Validation.

[96] Pantke U., Volk T., Schmutzler M., Kox W.J., Sitte N., Grune T. (1999). Oxidized proteins as a marker of oxidative stress during coronary heart surgery. Free Radic. Biol. Med..

[97] Pedersen-Lane J.H., Zurier R.B., Lawrence D.A. (2007). Analysis of the thiol status of peripheral blood leukocytes in rheumatoid arthritis patients. J. Leukoc. Biol..

[98] Peluffo G., Radi R. (2007). Biochemistry of protein tyrosine nitration in cardiovascular pathology. Cardiovasc. Res..

[99] Frijhoff J., Winyard P.G., Zarkovic N., Davies S.S., Stocker R., Cheng D., Knight A.R., Taylor E.L., Oettrich J., Ruskovska T. (2015). Clinical Relevance of Biomarkers of Oxidative Stress. Antioxid. Redox Signal..

[100] Winterbourn C.C. (2014). The challenges of using fluorescent probes to detect and quantify specific reactive oxygen species in living cells. Biochim. Biophys. Acta.

[101] Dikalov S.I., Harrison D.G. (2014). Methods for detection of mitochondrial and cellular reactive oxygen species. Antioxid. Redox Signal..

[102] Gomes A., Fernandes E., Lima J.L. (2005). Fluorescence probes used for detection of reactive oxygen species. J. Biochem. Biophys. Methods.

[103] Wakita C., Honda K., Shibata T., Akagawa M., Uchida K. (2011). A method for detection of 4-hydroxy-2-nonenal adducts in proteins. Free Radic. Biol. Med..

[104] Milne G.L., Sanchez S.C., Musiek E.S., Morrow J.D. (2007). Quantification of F2-isoprostanes as a biomarker of oxidative stress. Nat. Protoc..

[105] Il’yasova D., Kinev A., Melton C.D., Davis F.G. (2014). Donor-specific cell-based assays in studying sensitivity to low-dose radiation: A population-based perspective. Front. Public Health.

[106] Broedbaek K., Siersma V., Henriksen T., Weimann A., Petersen M., Andersen J.T., Jimenez-Solem E., Hansen L.J., Henriksen J.E., Bonnema S.J. (2015). Urinary markers of nucleic acid oxidation and cancer in type 2 diabetes. Redox Biol..

[107] Levine R.L., Garland D., Oliver C.N., Amici A., Climent I., Lenz A.G., Ahn B.W., Shaltiel S., Stadtman E.R. (1990). Determination of carbonyl content in oxidatively modified proteins. Methods Enzym..

[108] Buss H., Chan T.P., Sluis K.B., Domigan N.M., Winterbourn C.C. (1997). Protein carbonyl measurement by a sensitive ELISA method. Free Radic. Biol. Med..

[109] Keller R.J., Halmes N.C., Hinson J.A., Pumford N.R. (1993). Immunochemical detection of oxidized proteins. Chem. Res. Toxicol..

[110] Delgado-Andrade C. (2016). Carboxymethyl-lysine: Thirty years of investigation in the field of AGE formation. Food Funct..

[111] Vistoli G., De Maddis D., Cipak A., Zarkovic N., Carini M., Aldini G. (2013). Advanced glycoxidation and lipoxidation end products (AGEs and ALEs): An overview of their mechanisms of formation. Free Radic. Res..

[112] Colzani M., Aldini G., Carini M. (2013). Mass spectrometric approaches for the identification and quantification of reactive carbonyl species protein adducts. J. Proteom...

[113] Ashraf J.M., Ahmad S., Choi I., Ahmad N., Farhan M., Tatyana G., Shahab U. (2015). Recent advances in detection of AGEs: Immunochemical, bioanalytical and biochemical approaches. IUBMB Life.

[114] Da Moura S.C., Webb M., Waller H., Khunti K., Davies M. (2017). Skin autofluorescence, a non-invasive marker of advanced glycation end products: Clinical relevance and limitations. Postgrad. Med. J..

[115] Verhoye E., Langlois M.R., Asklepios I. (2009). Circulating oxidized low-density lipoprotein: A biomarker of atherosclerosis and cardiovascular risk?. Clin. Chem. Lab. Med..

[116] Holvoet P., De Keyzer D., Jacobs D.R. (2008). Oxidized LDL and the metabolic syndrome. Future Lipidol..

[117] Serafini M., Villano D., Spera G., Pellegrini N. (2006). Redox molecules and cancer prevention: The importance of understanding the role of the antioxidant network. Nutr. Cancer.

[118] Laurindo F.R., Pescatore L.A., Fernandes Dde C. (2012). Protein disulfide isomerase in redox cell signaling and homeostasis. Free Radic. Biol. Med..

[119] Lu J., Holmgren A. (2014). The thioredoxin antioxidant system. Free Radic. Biol. Med..

[120] Pastore A., Mozzi A.F., Tozzi G., Gaeta L.M., Federici G., Bertini E., Lo Russo A., Mannucci L., Piemonte F. (2003). Determination of glutathionyl-hemoglobin in human erythrocytes by cation-exchange high-performance liquid chromatography. Anal. Biochem..

[121] Knasmuller S., Nersesyan A., Misik M., Gerner C., Mikulits W., Ehrlich V., Hoelzl C., Szakmary A., Wagner K.H. (2008). Use of conventional and -omics based methods for health claims of dietary antioxidants: A critical overview. Br. J. Nutr..

[122] Giamogante F., Marrocco I., Romaniello D., Eufemi M., Chichiarelli S., Altieri F. (2016). Comparative Analysis of the Interaction between Different Flavonoids and PDIA3. Oxid. Med. Cell Longev..

[123] Veglia F., Cighetti G., De Franceschi M., Zingaro L., Boccotti L., Tremoli E., Cavalca V. (2006). Age- and gender-related oxidative status determined in healthy subjects by means of OXY-SCORE, a potential new comprehensive index. Biomarkers.

[124] Vassalle C. (2008). An easy and reliable automated method to estimate oxidative stress in the clinical setting. Methods Mol. Biol..

[125] Clark A., Mach N. (2017). The Crosstalk between the Gut Microbiota and Mitochondria during Exercise. Front. Physiol..

[126] Aviello G., Knaus U.G. (2017). ROS in gastrointestinal inflammation: Rescue or Sabotage?. Br. J. Pharm..

[127] Mandal D., Fu P., Levine A.D. (2010). REDOX regulation of IL-13 signaling in intestinal epithelial cells: Usage of alternate pathways mediates distinct gene expression patterns. Cell Signal..

[128] Hecker L., Vittal R., Jones T., Jagirdar R., Luckhardt T.R., Horowitz J.C., Pennathur S., Martinez F.J., Thannickal V.J. (2009). NADPH oxidase-4 mediates myofibroblast activation and fibrogenic responses to lung injury. Nat. Med..

[129] Diebold I., Flugel D., Becht S., Belaiba R.S., Bonello S., Hess J., Kietzmann T., Gorlach A. (2010). The hypoxia-inducible factor-2alpha is stabilized by oxidative stress involving NOX4. Antioxid. Redox Signal..

[130] Cao S.T., Wang C.C., Wu H., Zhang Q.H., Jiao L.F., Hu C.H. (2018). Weaning disrupts intestinal antioxidant status, impairs intestinal barrier and mitochondrial function, and triggers mitophagy in piglets. J. Anim. Sci..

[131] Crakes K.R., Santos Rocha C., Grishina I., Hirao L.A., Napoli E., Gaulke C.A., Fenton A., Datta S., Arredondo J., Marco M.L. (2019). PPARalpha-targeted mitochondrial bioenergetics mediate repair of intestinal barriers at the host-microbe intersection during SIV infection. Proc. Natl. Acad. Sci. USA.

[132] Lupp C., Robertson M.L., Wickham M.E., Sekirov I., Champion O.L., Gaynor E.C., Finlay B.B. (2007). Host-mediated inflammation disrupts the intestinal microbiota and promotes the overgrowth of Enterobacteriaceae. Cell Host Microbe.

[133] Winter S.E., Keestra A.M., Tsolis R.M., Baumler A.J. (2010). The blessings and curses of intestinal inflammation. Cell Host Microbe.

[134] Perez S., Talens-Visconti R., Rius-Perez S., Finamor I., Sastre J. (2017). Redox signaling in the gastrointestinal tract. Free Radic. Biol. Med..

[135] Jimenez P., Piazuelo E., Sanchez M.T., Ortego J., Soteras F., Lanas A. (2005). Free radicals and antioxidant systems in reflux esophagitis and Barrett’s esophagus. World J. Gastroenterol..

[136] Fu X., Beer D.G., Behar J., Wands J., Lambeth D., Cao W. (2006). cAMP-response element-binding protein mediates acid-induced NADPH oxidase NOX5-S expression in Barrett esophageal adenocarcinoma cells. J. Biol. Chem..

[137] Hong J., Li D., Cao W. (2016). Rho Kinase ROCK2 Mediates Acid-Induced NADPH Oxidase NOX5-S Expression in Human Esophageal Adenocarcinoma Cells. PLoS ONE.

[138] Li D., Cao W. (2014). Role of intracellular calcium and NADPH oxidase NOX5-S in acid-induced DNA damage in Barrett’s cells and Barrett’s esophageal adenocarcinoma cells. Am. J. Physiol. Gastrointest. Liver Physiol..

[139] Zhang Q.B., Nakashabendi I.M., Mokhashi M.S., Dawodu J.B., Gemmell C.G., Russell R.I. (1996). Association of cytotoxin production and neutrophil activation by strains of Helicobacter pylori isolated from patients with peptic ulceration and chronic gastritis. Gut.

[140] Zhang Q.B., Dawodu J.B., Husain A., Etolhi G., Gemmell C.G., Russell R.I. (1997). Association of antral mucosal levels of interleukin 8 and reactive oxygen radicals in patients infected with Helicobacter pylori. Clin. Sci. (Lond.).

[141] Kawahara T., Kohjima M., Kuwano Y., Mino H., Teshima-Kondo S., Takeya R., Tsunawaki S., Wada A., Sumimoto H., Rokutan K. (2005). Helicobacter pylori lipopolysaccharide activates Rac1 and transcription of NADPH oxidase Nox1 and its organizer NOXO1 in guinea pig gastric mucosal cells. Am. J. Physiol. Cell Physiol..

[142] Chaturvedi R., Asim M., Barry D.P., Frye J.W., Casero R.A., Wilson K.T. (2014). Spermine oxidase is a regulator of macrophage host response to Helicobacter pylori: Enhancement of antimicrobial nitric oxide generation by depletion of spermine. Amino Acids.

[143] Colgan S.P., Taylor C.T. (2010). Hypoxia: An alarm signal during intestinal inflammation. Nat. Rev. Gastroenterol. Hepatol..

[144] Kruidenier L., Kuiper I., Lamers C.B., Verspaget H.W. (2003). Intestinal oxidative damage in inflammatory bowel disease: Semi-quantification, localization, and association with mucosal antioxidants. J. Pathol..

[145] Krieglstein C.F., Cerwinka W.H., Laroux F.S., Salter J.W., Russell J.M., Schuermann G., Grisham M.B., Ross C.R., Granger D.N. (2001). Regulation of murine intestinal inflammation by reactive metabolites of oxygen and nitrogen: Divergent roles of superoxide and nitric oxide. J. Exp. Med..

[146] Yao J., Wang J.Y., Liu L., Zeng W.S., Li Y.X., Xun A.Y., Zhao L., Jia C.H., Feng J.L., Wei X.X. (2011). Polydatin ameliorates DSS-induced colitis in mice through inhibition of nuclear factor-kappaB activation. Planta Med..

[147] Singer I.I., Kawka D.W., Scott S., Weidner J.R., Mumford R.A., Riehl T.E., Stenson W.F. (1996). Expression of inducible nitric oxide synthase and nitrotyrosine in colonic epithelium in inflammatory bowel disease. Gastroenterology.

[148] Alzoghaibi M.A. (2013). Concepts of oxidative stress and antioxidant defense in Crohn’s disease. World J. Gastroenterol..

[149] Zane A.C., Reiter D.A., Shardell M., Cameron D., Simonsick E.M., Fishbein K.W., Studenski S.A., Spencer R.G., Ferrucci L. (2017). Muscle strength mediates the relationship between mitochondrial energetics and walking performance. Aging Cell.

[150] Che Man R., Sulaiman N., Ishak M.F., Idrus B.T.R., Abdul Rahman M.R., Yazid M.D. (2020). The Effects of Pro-Inflammatory and Anti-Inflammatory Agents for the Suppression of Intimal Hyperplasia: An Evidence-Based Review. Int. J. Env. Res. Public Health.

[151] Vona R., Ascione B., Malorni W., Straface E. (2018). Mitochondria and Sex-Specific Cardiac Function. Adv. Exp. Med. Biol..

[152] Mills E., O’Neill L.A. (2014). Succinate: A metabolic signal in inflammation. Trends Cell Biol..

[153] Trian T., Benard G., Begueret H., Rossignol R., Girodet P.O., Ghosh D., Ousova O., Vernejoux J.M., Marthan R., Tunon-de-Lara J.M. (2007). Bronchial smooth muscle remodeling involves calcium-dependent enhanced mitochondrial biogenesis in asthma. J. Exp. Med..

[154] Hoffmann R.F., Zarrintan S., Brandenburg S.M., Kol A., de Bruin H.G., Jafari S., Dijk F., Kalicharan D., Kelders M., Gosker H.R. (2013). Prolonged cigarette smoke exposure alters mitochondrial structure and function in airway epithelial cells. Respir. Res..

[155] Schiffrin E.L. (2008). Oxidative stress, nitric oxide synthase, and superoxide dismutase: A matter of imbalance underlies endothelial dysfunction in the human coronary circulation. Hypertension.

[156] Pirillo A., Norata G.D., Catapano A.L. (2013). LOX-1, OxLDL, and atherosclerosis. Mediat. Inflamm..

[157] Gonzalez A., Sarna S.K. (2001). Different types of contractions in rat colon and their modulation by oxidative stress. Am. J. Physiol. Gastrointest. Liver Physiol..

[158] Shi X.Z., Lindholm P.F., Sarna S.K. (2003). NF-kappa B activation by oxidative stress and inflammation suppresses contractility in colonic circular smooth muscle cells. Gastroenterology.

[159] Singh J., Kumar S., Rattan S. (2015). Bimodal effect of oxidative stress in internal anal sphincter smooth muscle. Am. J. Physiol. Gastrointest. Liver Physiol..

[160] Al-Shboul O., Mustafa A. (2015). Effect of oxidative stress on Rho kinase II and smooth muscle contraction in rat stomach. Can. J. Physiol. Pharm..

[161] Rao G.N., Berk B.C. (1992). Active oxygen species stimulate vascular smooth muscle cell growth and proto-oncogene expression. Circ. Res..

[162] Fiorani M., Cantoni O., Tasinato A., Boscoboinik D., Azzi A. (1995). Hydrogen peroxide-and fetal bovine serum-induced DNA synthesis in vascular smooth muscle cells: Positive and negative regulation by protein kinase C isoforms. Biochim. Biophys. Acta.

[163] Song H.J., Lee T.S., Jeong J.H., Min Y.S., Shin C.Y., Sohn U.D. (2005). Hydrogen peroxide-induced extracellular signal-regulated kinase activation in cultured feline ileal smooth muscle cells. J. Pharm. Exp..

[164] Scirocco A., Pallotta L., Rengo M., Ignazzi A., Carabotti M., Cicenia A., Vona R., Chirletti P., Maselli M.A., Donghia R. (2018). Myogenic oxidative imbalance interferes with antral motility in obese subjects. Dig. Liver Dis..

[165] Rani V., Deep G., Singh R.K., Palle K., Yadav U.C. (2016). Oxidative stress and metabolic disorders: Pathogenesis and therapeutic strategies. Life Sci..

[166] Savini I., Catani M.V., Evangelista D., Gasperi V., Avigliano L. (2013). Obesity-associated oxidative stress: Strategies finalized to improve redox state. Int. J. Mol. Sci..

[167] Guarino M.P., Cicala M., Putignani L., Severi C. (2016). Gastrointestinal neuromuscular apparatus: An underestimated target of gut microbiota. World J. Gastroenterol..

[168] Simmonds N.J., Allen R.E., Stevens T.R., Van Someren R.N., Blake D.R., Rampton D.S. (1992). Chemiluminescence assay of mucosal reactive oxygen metabolites in inflammatory bowel disease. Gastroenterology.

[169] Hotta Y., Uchiyama K., Takagi T., Kashiwagi S., Nakano T., Mukai R., Toyokawa Y., Yasuda T., Ueda T., Suyama Y. (2018). Transforming growth factor beta1-induced collagen production in myofibroblasts is mediated by reactive oxygen species derived from NADPH oxidase 4. Biochem. Biophys. Res. Commun..

[170] Tattoli I., Petitta C., Scirocco A., Ammoscato F., Cicenia A., Severi C. (2012). Microbiota, innate immune system, and gastrointestinal muscle: Ongoing studies. J. Clin. Gastroenterol..

[171] Scirocco A., Matarrese P., Petitta C., Cicenia A., Ascione B., Mannironi C., Ammoscato F., Cardi M., Fanello G., Guarino M.P. (2010). Exposure of Toll-like receptors 4 to bacterial lipopolysaccharide (LPS) impairs human colonic smooth muscle cell function. J. Cell Physiol..

[172] Matarrese P., Petitta C., Scirocco A., Ascione B., Ammoscato F., Di Natale G., Anastasi E., Marconi M., Chirletti P., Malorni W. (2012). Antioxidants counteract lipopolysaccharide-triggered alterations of human colonic smooth muscle cells. Free Radic. Biol. Med..

[173] Wang G., Jacquet L., Karamariti E., Xu Q. (2015). Origin and differentiation of vascular smooth muscle cells. J. Physiol..

[174] Wall V.Z., Bornfeldt K.E. (2014). Arterial smooth muscle. Arter. Thromb. Vasc. Biol..

[175] Savoia C., Sada L., Zezza L., Pucci L., Lauri F.M., Befani A., Alonzo A., Volpe M. (2011). Vascular inflammation and endothelial dysfunction in experimental hypertension. Int. J. Hypertens.

[176] Steyers C.M., Miller F.J. (2014). Endothelial dysfunction in chronic inflammatory diseases. Int. J. Mol. Sci..

[177] Guarino M.P., Barbara G., Cicenia A., Altomare A., Barbaro M.R., Cocca S., Scirocco A., Cremon C., Emerenziani S., Stanghellini V. (2017). Supernatants of irritable bowel syndrome mucosal biopsies impair human colonic smooth muscle contractility. Neurogastroenterol. Motil..

[178] Brookes M.J., Green J.R. (2004). Maintenance of remission in Crohn’s disease: Current and emerging therapeutic options. Drugs.

[179] Burger M., Schmidt C., Teich N., Stallmach A. (2015). Medical Therapy of Active Ulcerative Colitis. Viszeralmedizin.

[180] Serban D.E. (2015). Microbiota in Inflammatory Bowel Disease Pathogenesis and Therapy: Is It All About Diet?. Nutr. Clin. Pract..

[181] Tian T., Wang Z., Zhang J. (2017). Pathomechanisms of Oxidative Stress in Inflammatory Bowel Disease and Potential Antioxidant Therapies. Oxid. Med. Cell Longev..

[182] Zhang X., Jiang A., Qi B., Ma Z., Xiong Y., Dou J., Wang J. (2015). Resveratrol Protects against Helicobacter pylori-Associated Gastritis by Combating Oxidative Stress. Int. J. Mol. Sci..

[183] Nunes S., Danesi F., Del Rio D., Silva P. (2018). Resveratrol and inflammatory bowel disease: The evidence so far. Nutr. Res. Rev..

[184] Vezza T., Rodriguez-Nogales A., Algieri F., Utrilla M.P., Rodriguez-Cabezas M.E., Galvez J. (2016). Flavonoids in Inflammatory Bowel Disease: A Review. Nutrients.

[185] De Filippis D., Iuvone T., Esposito G., Steardo L., Arnold G.H., Paul A.P., De Man Joris G., De Winter Benedicte Y. (2008). Melatonin reverses lipopolysaccharide-induced gastro-intestinal motility disturbances through the inhibition of oxidative stress. J. Pineal Res..

[186] Zulueta A., Caretti A., Signorelli P., Ghidoni R. (2015). Resveratrol: A potential challenger against gastric cancer. World J. Gastroenterol..

[187] Forman H.J., Davies K.J., Ursini F. (2014). How do nutritional antioxidants really work: Nucleophilic tone and para-hormesis versus free radical scavenging in vivo. Free Radic. Biol. Med..

[188] Boyanapalli S.S., Paredes-Gonzalez X., Fuentes F., Zhang C., Guo Y., Pung D., Saw C.L., Kong A.N. (2014). Nrf2 knockout attenuates the anti-inflammatory effects of phenethyl isothiocyanate and curcumin. Chem. Res. Toxicol..

[189] Soetikno V., Sari F.R., Lakshmanan A.P., Arumugam S., Harima M., Suzuki K., Kawachi H., Watanabe K. (2013). Curcumin alleviates oxidative stress, inflammation, and renal fibrosis in remnant kidney through the Nrf2-keap1 pathway. Mol. Nutr. Food Res..

[190] Ding Y., Chen M., Wang M., Wang M., Zhang T., Park J., Zhu Y., Guo C., Jia Y., Li Y. (2014). Neuroprotection by acetyl-11-keto-beta-Boswellic acid, in ischemic brain injury involves the Nrf2/HO-1 defense pathway. Sci. Rep..

[191] Khan I., Samson S.E., Grover A.K. (2017). Antioxidant Supplements and Gastrointestinal Diseases: A Critical Appraisal. Med. Princ. Pract..

[192] Taylor R.A., Leonard M.C. (2011). Curcumin for inflammatory bowel disease: A review of human studies. Altern Med. Rev..

[193] Baliga M.S., Joseph N., Venkataranganna M.V., Saxena A., Ponemone V., Fayad R. (2012). Curcumin, an active component of turmeric in the prevention and treatment of ulcerative colitis: Preclinical and clinical observations. Food Funct..

[194] Hanasaki Y., Ogawa S., Fukui S. (1994). The correlation between active oxygens scavenging and antioxidative effects of flavonoids. Free Radic. Biol. Med..

[195] Brown J.E., Khodr H., Hider R.C., Rice-Evans C.A. (1998). Structural dependence of flavonoid interactions with Cu2+ ions: Implications for their antioxidant properties. Biochem. J..

[196] Buettner G.R. (1993). The pecking order of free radicals and antioxidants: Lipid peroxidation, alpha-tocopherol, and ascorbate. Arch. Biochem. Biophys..

[197] Dima C., Assadpour E., Dima S., Jafari S.M. (2020). Nutraceutical nanodelivery; an insight into the bioaccessibility/bioavailability of different bioactive compounds loaded within nanocarriers. Crit. Rev. Food Sci. Nutr..

[198] Bertoni S., Machness A., Tiboni M., Bartolo R., Santos H.A. (2020). Reactive oxygen species responsive nanoplatforms as smart drug delivery systems for gastrointestinal tract targeting. Biopolymers.

[199] Vaiserman A., Koliada A., Zayachkivska A., Lushchak O. (2019). Nanodelivery of Natural Antioxidants: An Anti-aging Perspective. Front. Bioeng. Biotechnol..

[200] Sharma M., Sharma S., Wadhwa J. (2019). Improved uptake and therapeutic intervention of curcumin via designing binary lipid nanoparticulate formulation for oral delivery in inflammatory bowel disorder. Artif. Cells Nanomed. Biotechnol..

[201] Oskouie M.N., Aghili Moghaddam N.S., Butler A.E., Zamani P., Sahebkar A. (2019). Therapeutic use of curcumin-encapsulated and curcumin-primed exosomes. J. Cell Physiol..

[202] Guerrero S., Inostroza-Riquelme M., Contreras-Orellana P., Diaz-Garcia V., Lara P., Vivanco-Palma A., Cardenas A., Miranda V., Robert P., Leyton L. (2018). Curcumin-loaded nanoemulsion: A new safe and effective formulation to prevent tumor reincidence and metastasis. Nanoscale.

[203] Ventola C.L. (2017). Progress in Nanomedicine: Approved and Investigational Nanodrugs. Pharm. Ther..

[204] Li C.W., Li L.L., Chen S., Zhang J.X., Lu W.L. (2020). Antioxidant Nanotherapies for the Treatment of Inflammatory Diseases. Front. Bioeng. Biotechnol..

[205] Ahmed O.A.A., Fahmy U.A., Bakhaidar R., El-Moselhy M.A., Okbazghi S.Z., Ahmed A.F., Hammad A.S.A., Alhakamy N.A. (2020). Omega-3 Self-Nanoemulsion Role in Gastroprotection against Indomethacin-Induced Gastric Injury in Rats. Pharmaceutics.

